# A DNA barcode reference library for Swiss butterflies and forester moths as a tool for species identification, systematics and conservation

**DOI:** 10.1371/journal.pone.0208639

**Published:** 2018-12-21

**Authors:** Jessica Litman, Yannick Chittaro, Stefan Birrer, Christophe Praz, Emmanuel Wermeille, Markus Fluri, Thomas Stalling, Sarah Schmid, Sofia Wyler, Yves Gonseth

**Affiliations:** 1 Museum of Natural History of Neuchâtel, Neuchâtel, Switzerland; 2 info fauna – CSCF, Neuchâtel, Switzerland; 3 Hintermann & Weber AG, Reinach, Switzerland; 4 Biodiversity Monitoring Switzerland, Bern, Switzerland; 5 Laboratory of Evolutive Entomology, Institute of Biology, University of Neuchâtel, Neuchâtel, Switzerland; 6 Freelance biologist, Villiers, Switzerland; 7 Department of Computational Biology, University of Lausanne, Lausanne, Switzerland; Nanjing Agricultural University, CHINA

## Abstract

Butterfly monitoring and Red List programs in Switzerland rely on a combination of observations and collection records to document changes in species distributions through time. While most butterflies can be identified using morphology, some taxa remain challenging, making it difficult to accurately map their distributions and develop appropriate conservation measures. In this paper, we explore the use of the DNA barcode (a fragment of the mitochondrial gene COI) as a tool for the identification of Swiss butterflies and forester moths (Rhopalocera and Zygaenidae). We present a national DNA barcode reference library including 868 sequences representing 217 out of 224 resident species, or 96.9% of Swiss fauna. DNA barcodes were diagnostic for nearly 90% of Swiss species. The remaining 10% represent cases of para- and polyphyly likely involving introgression or incomplete lineage sorting among closely related taxa. We demonstrate that integrative taxonomic methods incorporating a combination of morphological and genetic techniques result in a rate of species identification of over 96% in females and over 98% in males, higher than either morphology or DNA barcodes alone. We explore the use of the DNA barcode for exploring boundaries among taxa, understanding the geographical distribution of cryptic diversity and evaluating the status of purportedly endemic taxa. Finally, we discuss how DNA barcodes may be used to improve field practices and ultimately enhance conservation strategies.

## Introduction

Butterflies, particularly sensitive to changes in the environment and thus often used as indicators of habitat quality, are among the most closely monitored insects. Dramatic losses have been documented throughout Europe [1–6] and North America [7–9]. The European Environment Agency reported an average decline of 30% in European grassland butterfly abundance between 1990 and 2015 [4]. Such losses, only quantifiable with observations and collection records spanning many years, highlight the importance of vigilant monitoring programs and the need for rigorous and immediate conservation measures.

In order to document trends in butterfly species richness and distributions, the Swiss Federal Office for the Environment (FOEN) hosts a longstanding national monitoring program, Biodiversity Monitoring Switzerland (BDM), which is part of a long-term strategy to track changes in biodiversity patterns and to publish and update red lists of threatened species. The data gathered by the BDM, complemented by targeted collecting carried out by info fauna - CSCF (The Swiss Faunistic Records Center), observations made by professional and volunteer naturalists and data gathered from museum collections, culminated in the publication of an updated Red List for the Rhopalocera and Zygaenidae (the butterflies and forester moths, hereafter the diurnal Lepidoptera) of Switzerland [10].

While the diurnal Lepidoptera of Switzerland and their distributions are well known, comparatively little is known about their genetic diversity. Within species, genetic diversity is critical to the maintenance of resilient populations that are able to adapt in shifting environmental landscapes, thus improving population persistence through time [11,12]. Understanding the geographical distribution of intraspecific genetic diversity is thus fundamental to the development of conservation action plans that target not only taxa but also genetic variation within taxa.

The DNA "barcode", a short, variable fragment of the mitochondrial gene cytochrome c oxidase subunit I (COI), has been successfully used as a tool for the identification of a broad range of animal taxa, including European Lepidoptera [13–20]. It is also used as a means of identifying potential cases of cryptic diversity, hybridization and incomplete lineage sorting [18,19]. Extensive barcode libraries exist for butterflies from other European countries, including Spain, Romania, Finland, Austria, northern Italy and Germany, yet Swiss genetic data for this group remains scant [13–15,17,18,20,21]. Switzerland, representing a major contact zone for many species and a crossroads for multiple biogeographic regions, represents a key part of the genetic diversity that exists across the continent.

The SwissBOL (Swiss Barcode of Life) Butterfly project was thus launched in 2013 to obtain baseline genetic data for Swiss diurnal Lepidoptera, to understand the geographical distribution of this diversity and to ultimately use this data to hone conservation strategies. In this article, we present a COI barcode library representing 217 species of resident butterflies and forester moths sampled across Switzerland, for a total of 96.9% of the country's species. We evaluate the utility of the barcode as a tool for Swiss species identification, for highlighting potential cryptic diversity and examining boundaries among taxa in groups that remain difficult to distinguish using morphology or ecology alone. We examine Swiss butterfly barcodes in the context of existing European sequences to assess the status of purportedly endemic taxa. Finally, we discuss how DNA barcodes can be used to improve field practices, update species distribution maps, fine-tune conservation measures and formulate recommendations on the potential use of DNA barcoding to improve species detection in the ongoing BDM program in Switzerland.

## Materials and methods

### Sampling strategy

**Fig 1:**
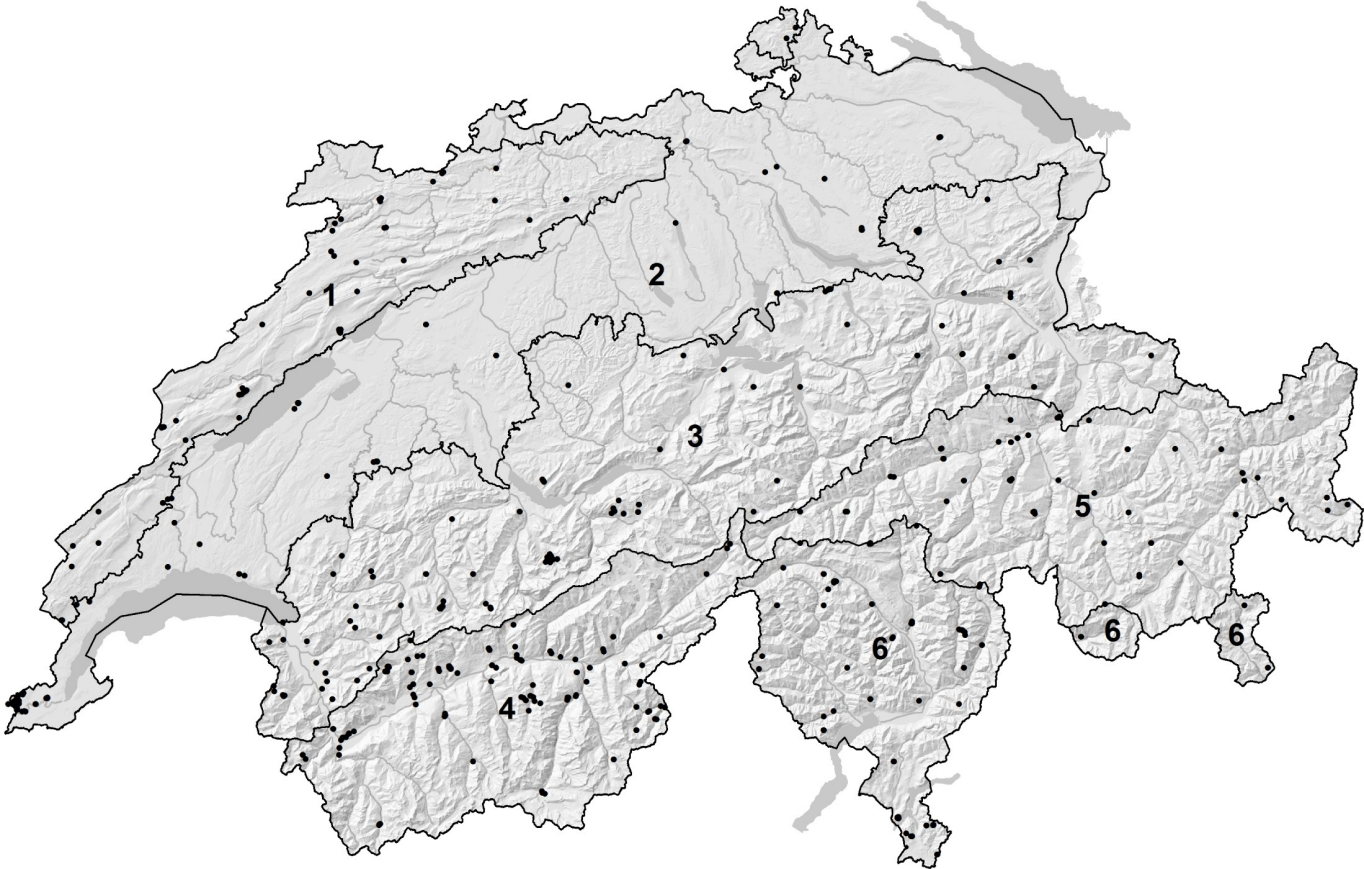
Biogeographic regions of Switzerland. 1. Jura; 2. Plateau; 3. Northern foothills of the Alps; 4. Western central Alps; 5. Eastern central Alps; and 6. Southern foothills of the Alps. Points represent the collection localities of all specimens sequenced during the course of this project.

Switzerland is divided into six biogeographic regions [21] (Fig 1). Each region exhibits a distinct combination of ecological, topological and climatological attributes and is considered a unique biogeographical entity. Our goal was to sample at least one individual per species from each of the biogeographic regions where the species was known to occur, in order to sample as broadly as possible from the distribution of each species. Individuals were netted, killed by freezing and later pinned and labeled (S1 Table). All specimens except one are deposited in the entomological collection of the Museum of Natural History of Neuchâtel, Switzerland. A leg from a single individual of *Coenonympha tullia* (sample ID GBIFCH-BOL_LEPAA_0906), sampled from a small, highly localized population in the Bernese Oberland, was taken in the field and the individual subsequently released. Localities are reported here at a scale of 10 km^2^. For more precise locality information, the second author may be contacted directly.

### Collecting permits

Field surveys coordinated by Info fauna – CSCF (www.cscf.ch), as well as species monitoring programs carried out by Biodiversity Monitoring Switzerland (www.biodiversitymonitoring.ch), are complementary programs both financed by the Swiss Confederation. The data gathered during the course of these projects are used in the preparation of national Red Lists and represent a valuable source of information for the definition of regional priorities for the conservation of species and their habitats. Furthermore, these data contribute to the systematic inventory of the fauna of Switzerland.

In Switzerland, collecting permits are obtained not at the federal level but rather via the individual cantons (Swiss states). There are 26 cantons in total, each with its own particular legislation concerning the collection of specimens in the field. Every year, those entities responsible for the field surveys and monitoring programs mentioned in the paragraph above organize a grouped request for collecting permits from each individual canton, for each individual collector. Authorizations are always specific to an individual person and are valid for a limited amount of time and within a specific canton. The collectors that provided specimens for this project are listed in the “Acknowledgments” section. Every specimen used in this project was sequenced within the context of the Swiss Barcode of Life (SwissBOL) project, a national initiative financed by the Swiss Federal Office for the Environment (OFEV) to obtain DNA sequences for Swiss species. All specimens were collected with the proper authorizations as provided by the appropriate cantonal authorities.

### Species list

Two hundred twenty-six species of butterflies and zygaenid moths were considered residents of Switzerland on the national Red List of Rhopalocera and Zygaenidae [10]. The taxonomic choices used on the Red List were based on a previously published national checklist [22]. We thus used the Swiss Red List as our reference, with the following modifications. Three species assessed as regionally extinct in Switzerland according to IUCN criteria were not included for the purposes of our study: *Carcharodus baeticus* (Rambur, 1839), not observed in Switzerland since 1954; *Aglaope infausta* (Linnaeus, 1767), not observed since 1974; and *Arethusana arethusa* (Denis & Schiffermüller, 1775), not observed since 1974. *Argynnis pandora* (Denis & Schiffermüller, 1775) was considered as an irregular migrator to Switzerland on the Red List and *Cacyreus marshalli* Butler, 1898 as an introduced species. These taxa, however, now have established populations in Switzerland and were thus considered residents for the purposes of our study.

Moreover, we consider *Phengaris alcon* (Denis & Schiffermüller, 1775) and *P*. *rebeli* (Hirschke, 1904) as two ecotypes of *P*. *alcon*, based on the results of a recent study [23]. We also updated the taxonomy of certain species based on changes proposed in a number of recent publications, including those concerning members of the families Lycaenidae and Nymphalidae [24,25], as well as those proposed in the German national checklist [26]. We thus consider the presence of 224 species of diurnal Lepidoptera in total (S2 Table).

### Morphological identification of specimens

Specimens were identified based on an examination of wing pattern and, for species where wing pattern is considered insufficient for species identification, the preparation and examination of genitalia. In an attempt to ensure the accuracy of morphology-based identifications, the identifications of specimens representing difficult species were verified by an authority in Swiss butterfly taxonomy (S1 Table).

### DNA extraction, PCR and sequencing

Genomic DNA was extracted from one or several legs, using two commercial kits *–* DNeasy Blood & Tissue Kit (Qiagen) and NucleoSpin 96 Tissue (Macherey-Nagel) and following the supplier’s instructions by eluting DNA to a final volume of 50 μL. The standard animal barcode region (a 658 bp fragment at the 5*'* end of the mitochondrial cytochrome c oxidase subunit I) was amplified using either LCO1490 and HCO2198 or LepF1 and LepR1 primers. PCRs were performed in 20 μL total volume with 0.60 U Taq (Roche), 2 μL of the 10X buffer containing 20 mM MgCl2, 0.8 μL of each primer (10 mM), 0.4 μl of a mix containing 10 mM of each dNTP (Roche) and 0.8 μL template DNA of unknown concentration. The PCR program comprised an initial denaturation phase at 95°C for 5 min, followed by 35 cycles at 95°C for 40 s, at 42°C for 45 s and at 72°C for 1 min, with a final elongation step at 72°C for 8 min. Positive PCR products were then directly bi-directionally sequenced on an ABI 3031 automated sequencer (Applied Biosystems) at the University of Geneva, using the same primers used for amplification and following the manufacturer’s protocol.

### Sequence alignment and analyses

Chromatograms were edited using Geneious [27] and Sequencher [28], aligned using MAFFT [29] and then given a final error check by visualizing the resulting alignment in Mesquite [30]. All sequences are available on BOLD Systems under the project “LEPAA” (www.boldsystems.org). Unique specimen numbers and BOLD process ID numbers are given in S1 Table. Neighbor-joining (NJ) analyses were performed using the BOLD v4 platform [31] and NJ bootstrap analyses were performed in PAUP, using a K2P model of substitution [32]. Maximum likelihood analyses were performed using RAxML v8.2.10 [33] as provided by the CIPRES server [34]. One thousand bootstrap replicates were run using the rapid bootstrapping algorithm, followed by a search for the best-scoring maximum likelihood tree based on the original DNA alignment. Mean and maximum intraspecific divergence and minimum genetic distance to a nearest neighbor were calculated under a K2P model of substitution and using the “MUSCLE” alignment option [35] on the BOLD v4 platform. This automated alignment was double-checked for accuracy and found to be identical to the alignment using the MAFFT + Mesquite method described above. A K2P corrected distance of zero to a nearest-neighbor was interpreted as a shared (identical) barcode.

## Results

A total of 868 DNA barcode sequences were obtained for 217 resident species representing the families Hesperiidae, Nymphalidae, Lycaenidae, Papilionidae, Pieridae, Riodinidae and Zygaenidae, for a total of 96.9% of Swiss fauna (S1 Fig, S1 Table). *Lampides boeticus*, a non-resident migratory species occasionally observed in Switzerland, was also sequenced, bringing the total number of species sequenced up to 218. We were unable to obtain sequences for the following seven species (marked in red in S2 Table): *Adscita mannii*, *Aricia nicias*, *Coenonympha hero*, *Coenonympha oedippus*, *Jordanita chloros*, *Melitaea britomartis* and *Pyrgus cirsii*. Six of these species are evaluated as critically endangered on the national Red List (*A*. *mannii*, *C*. *hero*, *C*. *oedippus*, *J*. *chloros*, *M*. *britomartis* and *P*. *cirsii*). Of these six, three have not been observed for at least ten years and may already have disappeared from Switzerland (*C*. *oedippus*, *J*. *chloros* and *P*. *cirsii*). Four specimens on average were sequenced per species. Maximum intraspecific divergence ranged from 0.0% to 5.63%, with an average of 0.59% (average based on species represented by at least two barcode sequences). Minimum genetic distance to a nearest non-conspecific terminal (nearest neighbor) ranged from 0.0% to 11.63%, with an average of 4.48%.

The results of our NJ analysis show that 174 species (80.2%) form monophyletic barcode clusters, allowing for their unambiguous identification (S1 Fig). Twenty-three additional species were represented by single sequences; of these, the sequences for two species, *Erebia nivalis* and *Pyrgus accreta*, were identical to those of other Swiss species and are discussed below in the section on “Para- and polyphyletic species”. The remaining 21 singletons were queried against other central European sequences from the BOLD database as a means of assessing the utility of the barcode as a tool to identify these species. Twenty species were unambiguously matched to the correct species; only *Erebia styx*, matched to both *E*. *styx* and *E*. *stirius* (the latter absent from Switzerland), resulted in an ambiguous identification. Across Swiss samples, the DNA barcode was thus diagnostic for 195 out of 217 (89.9%) resident species. Here we define a “diagnostic” barcode as one that assigns an individual of a species to a barcode cluster consisting exclusively of other members of the same species, i.e. the “criterion of barcode clusters” as implemented in [13]. We thus do not consider sequences from mixed clusters as “diagnostic” because they do not allow for the unambiguous identification of new sequences from the same assemblages if those sequences are not identical to existing sequences (but see Discussion under section entitled “Para- and polyphyletic taxa” for further discussion).

### Para- and polyphyletic species

A total of twenty-two species (10.1%) in eight pairs and two trios were para- or polyphyletic at the sequence-level. Four species pairs (3.7%) were para- or polyphyletic without sharing barcodes: *Pyrgus malvae* – *P*. *malvoides*, *Adscita statices – A*. *alpina*, *Plebejus argyrognomon* – *P*. *idas* and *Erebia manto – E*. *bubastis*. Six species pairs or trios (6.5%) were para- or polyphyletic and contained individuals sharing barcodes with at least one other member of the same group: *Cupido osiris* – *C*. *minimus*, *Erebia tyndarus* – *E*. *arvernensis* – *E*. *nivalis*, *Erebia ligea* – *E*. *euryale*, *Coenonympha darwiniana* – *C*. *gardetta*, *Zygaena minos – Z*. *purpuralis* and *Pyrgus warrenensis* – *P*. *alveus* – *P*. *accreta*. The morphology-based identifications of all individuals emerging as part of either para- or polyphyletic clusters were carefully reviewed to ensure that the observed results were the result of genetic phenomena and not an artefact of species misidentification.

### Potential cases of cryptic diversity

Ten species (4.6%) were characterized by levels of intraspecific divergence over 2% and five species exhibited levels over 3%: *Zygaena lonicerae* (2.03%), *Limenitis camilla* (2.36%), *Aphantopus hyperantus* (2.39%), *Zygaena transalpina* (2.67%), *Plebejus argyrognomon* (2.82%), *Thymelicus lineola* (3.27%), *Thymelicus sylvestris* (3.31%), *Eumedonia eumedon* (4.43%), *Melitaea athalia*, which until recently was treated in the Swiss national database as conspecific with *M*. *nevadensis*, (5.31%) and *Zygaena filipendulae* (5.63%). *Erebia manto* exhibited a level of intraspecific divergence of 2.3% but was paraphyletic with respect to *Erebia bubastis*. For each species, chromatograms were re-examined visually as a means of assuring sequence quality.

### Utility of the barcode for identifying Swiss taxa

The morphological identification of species depends on the presence of a certain combination of “typical” species-specific characters. Information regarding the geographical locality or the altitude where a specimen was collected may also serve as a clue for species identification. For certain closely related, morphologically similar taxa, however, the absence of diagnostic characters may render species identification difficult or impossible, even more so at contact zones between species.

At the outset of this study, eight pairs of taxa were highlighted as problematic for morphological determination because species in these pairs are either difficult or impossible to separate in either one or both sexes in Switzerland: *Aricia agestis* – *A*. *artaxerxes* (cannot always be separated with certainty in either sex), *Boloria napaea* - *B*. *pales* (cannot be separated with certainty in males), *Coenonympha darwiniana* – *C*. *gardetta* (cannot always be separated with certainty in either sex in contact zones), *Colias hyale* – *C*. *alfacariensis* (cannot be separated with certainty in females; some males are also difficult), *Erebia tyndarus* – *E*. *arvernensis – E*. *nivalis* (cannot be separated with certainty in females; examination of genitalia necessary for males), *Hipparchia fagi – H*. *genava* (cannot be separated with certainty in females; examination of genitalia necessary for males), *Pieris bryoniae* – *P*. *napi* (cannot be separated with certainty in males) and *Zygaena romeo* – *Z*. *osterodensis* (examination of genitalia necessary for males and females but some specimens, both male and female, remain difficult) (Table 1). In Swiss biodiversity surveys, these species are sometimes not distinguished and may simply be treated as aggregates.

**Table 1:** Identification success rate using the DNA barcode, morphology (including wing pattern and/or examination of genitalia) and integrated methods (representing a combination of DNA barcodes and morphology).

	DNA barcode alone	Male morphology alone	Female morphology alone	Integrated methods males	Integrated methods females
Identification always possible	89.9%	92.6%	91.7%	98.2%	96.3%
Identification difficult in at least some cases	10.1% • *Pyrgus malvae* – *P*. *malvoides* • *Adscita statices* – *A*. *alpina* • *Plebejus argyrognomon* – *P*. *idas* • *Erebia manto* – *E*. *bubastis* • *Erebia tyndarus* – *E*. *arvernensis* – *E*. *nivalis* • *Erebia ligea* – *E*. *euryale* • *Coenonympha darwiniana* – *C*. *gardetta* • *Zygaena minos – Z*. *purpuralis* • *Pyrgus warrenensis* – *P*. *alveus* – *P*. *accreta* • *Cupido osiris – C*. *minimus*	7.4% • *Aricia artaxerxes – A*. *agestis* • *Boloria napaea – B*. *pales* • *Pieris bryoniae – P*. *napi* • *Pyrgus accreta – P*. *alveus* • *Zygaena romeo – Z*. *osterodensis* • *Coenonympha gardetta – C*. *darwiniana*, • *Colias hyale – C*. *alfacariensis* • *Melitaea athalia – M*. *nevadensis*	8.3% • *Aricia artaxerxes – A*. *agestis* • *Colias alfacariensis - C*. *hyale* • *Erebia tyndarus – E*. *arvernensis – E*. *nivalis* • *Hipparchia fagi – H*. *genava* • *Melitaea athalia – M*. *nevadensis* • *Pyrgus accreta – P*. *alveus – P*. *warrenensis* • *Zygaena romeo – Z*. *osterodensis* • *Coenonympha gardetta – C*. *darwiniana*	1.8% • *Pyrgus accreta – P*. *alveus* • *Coenonympha gardetta – C*. *darwiniana*	3.7% • *Erebia tyndarus – E*. *arvernensis – E*. *nivalis* • *Pyrgus accreta – P*. *alveus – P*. *warrenensis* • *Coenonympha gardetta – C*. *darwiniana*

*Colias hyale* and *C*. *alfacariensis*, for example, are sometimes identified simply as “*Colias hyale*-*alfacariensis*”. This solution bypasses the problem of assigning a definitive species name to specimens of uncertain identity and in some cases may represent the only “honest” solution to identifying certain specimens. It is, however, the practical equivalent of “lumping” closely related species together into a single entity, resulting in the loss of fine-scale resolution at the species level. One of our objectives was to test the performance of the DNA barcode for differentiating these pairs in order to enhance the resolution of biodiversity surveys.

The DNA barcode successfully distinguished six out of the eight problematic species groups mentioned above: *Aricia agestis* – *A*. *artaxerxes*, *Boloria napaea* – *B*. *pales*, *Colias hyale* – *C*. *alfacariensis*, *Hipparchia fagi* – *H*. *genava*, *Pieris bryoniae* - *P*. *napi*, and *Zygaena romeo* – *Z*. *osterodensis*. The two remaining species groups, *Coenonympha gardetta – C*. *darwiniana* and *Erebia tyndarus* – *E*. *arvernensis – E*. *nivalis*, are discussed in the section on “Para- and polyphyletic taxa” below. A recent study of Austrian butterflies [20] also successfully used the DNA barcode to differentiate *C*. *hyale* - *alfacariensis*, *B*. *pales - napaea* and *A*. *artaxerxes – agestis*. Another study of Romanian butterflies [13] used the barcode to separate *C*. *hyale* – *alfacariensis* and *A*. *artaxerxes – agestis*. Of particular interest for Switzerland was the success of the barcode for differentiating *Aricia artaxerxes – A*. *agestis* and *Zygaena romeo* and *Z*. *osterodensis*, each discussed in detail below.

## Discussion

Here we present the first comprehensive COI barcode library for Swiss diurnal Lepidoptera, thus providing baseline genetic data for identifying species, exploring taxonomic boundaries and documenting the geographic distribution of mitochondrial genetic diversity in Switzerland. DNA barcodes were amplified for 217 out of 224 species (96.9%) and were diagnostic for 195 of 217 species included in this study, a success rate of 89.9%. Our success rate is similar to those of other regional studies of European Lepidoptera implementing a definition of “diagnostic” similar to our own, despite the fact that these regions often differ significantly in size: 86% for Austrian butterflies [20], 90% for Romanian butterflies [13] and 93.9% for Iberian butterflies [18]. Our results demonstrate that integrated methods combining DNA barcoding with morphology-based methods increase the success rate of species identification to 96.3% in females and 98.2% in males (Table 1). This represents an improvement of 6.4% over the barcode alone and 4.6% over morphology alone for females and an improvement of 8.3% over the barcode alone and 5.6% over morphology alone for males (Table 1).

### DNA barcodes for discerning morphologically similar taxa

**Fig 2:**
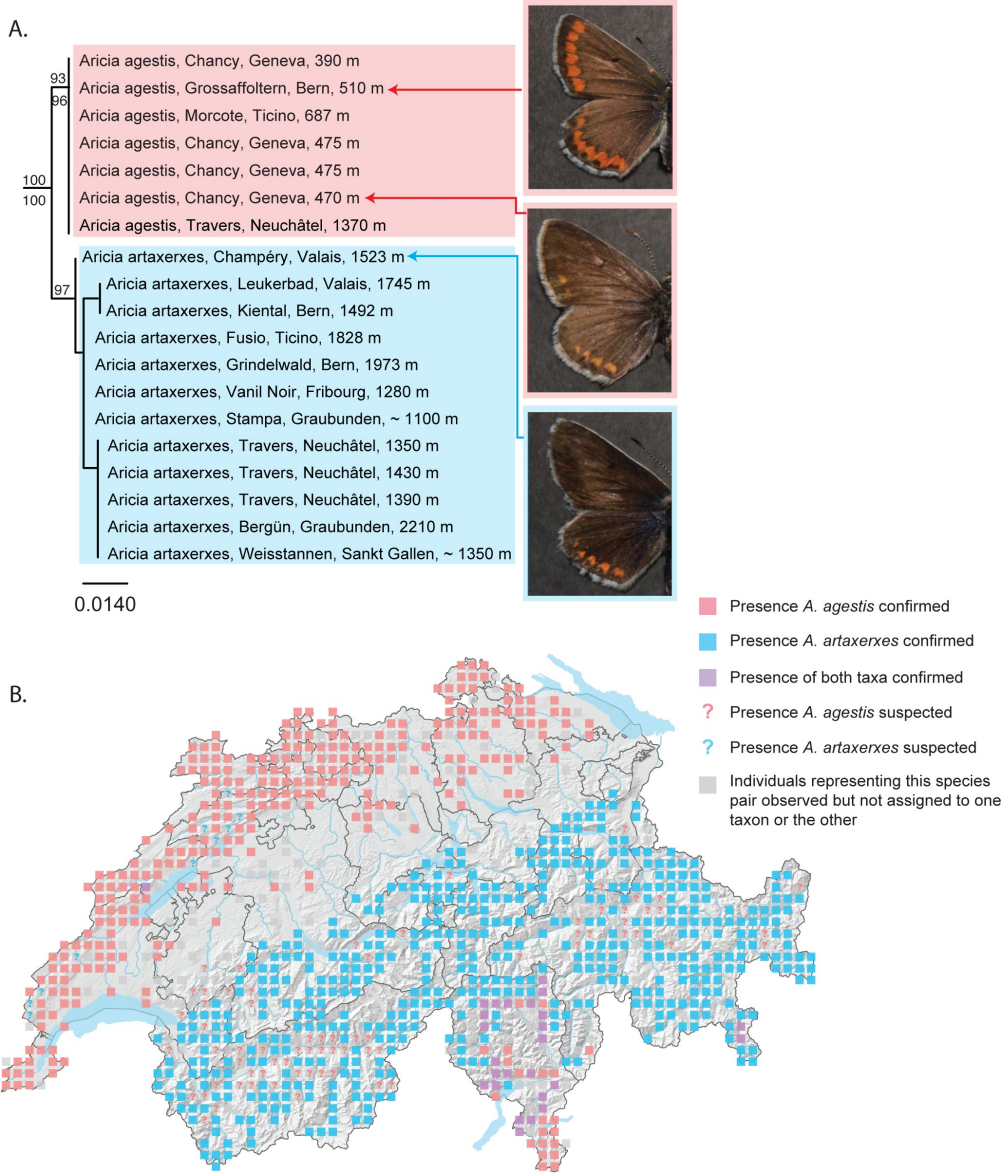
*Aricia agestis* and *A*. *artaxerxes* form reciprocally monophyletic barcode clusters. (A) NJ tree based on DNA barcodes for *Aricia agestis* and *A*. *artaxerxes*. Numbers above nodes are NJ bootstrap values over 50% as calculated in PAUP*. Numbers under nodes are maximum likelihood bootstrap values over 50% as calculated in RAxML. Images show variation in wing pattern commonly used to distinguish the two species. Vivid orange markings are strongly present on both fore- and hindwings in *A*. *agestis* (upper photo) and are weaker in *A*. *artaxerxes* (lower photo). Certain individuals of *A*. *agestis*, clearly clustering with other *A*. *agestis*, show wing patterns that are intermediate between the two taxa (middle photo). (B) Map of Switzerland showing distribution of *A*. *agestis* (pink squares) and *A*. *artaxerxes* (blue squares). Squares represent 5km^2^ quadrats. Localities where both species are found in the same quadrat are shown as purple squares. Localities where *A*. *agestis* is suspected to occur are shown as pink question marks. Localities where *A*. *artaxerxes* is suspected to occur are shown as blue question marks. Localities where individuals have been observed but not assigned to one taxon or the other are shown as grey squares. Note: Colored squares represent all data present in the national database. Certain populations shown on the map may represent historical populations that no longer exist.

*Aricia agestis* and *A*. *artaxerxes* are closely related species that are sometimes difficult to distinguish with certainty in both males and females [36], particularly in Central Europe. While the orange markings on the upper wing surfaces are typically brighter and more visible in *A*. *agestis* than in *A*. *artaxerxes*, intermediate forms can render identification difficult and no characters in either the male or female genitalia allow for the differentiation of these taxa. Due to the difficulties associated with identifying certain populations, their geographical distributions are not entirely clear. It is known that *A*. *agestis* occurs at low altitudes in the Jura, on the Plateau and in Ticino, while *A*. *artaxerxes* is present throughout the Alps. Populations at low elevations in the Alps and at higher elevations in the Jura, however, are difficult to assign to one taxon or the other. Certain populations at higher altitude in the Jura, for example, are darker and thus phenotypically more like *A*. *artaxerxes* but occur in a region historically associated with *A*. *agestis*. While *A*. *agestis* was previously considered bivoltine and *A*. *artaxerxes* univoltine, research has shown that voltinism is not an appropriate indicator of species identity and is most likely related to both altitudinal and latitudinal distribution [37]. Artificial rearings have shown hybrids to be reproductively inviable [36]. Our results demonstrate that *Aricia agestis* and *A*. *artaxerxes* emerge as two reciprocally monophyletic clades exhibiting a minimum genetic distance of 1.46% (Fig 2). The barcode is thus able to successfully differentiate these taxa in Switzerland, as appears to be the case for other countries in Europe, including Romania and Austria [13, 20]. Our results also confirm the presence of *A*. *artaxerxes* in the Jura. Furthermore, the discovery of one specimen of *A*. *agestis* and one of *A*. *artaxerxes* one kilometer apart in the Jura Mountains (canton of Neuchâtel) and at approximately the same elevation (~ 1400 m) demonstrate that these taxa exist in close proximity to one another and maintain genetic differentiation in sympatry (Fig 2).

**Fig 3:**
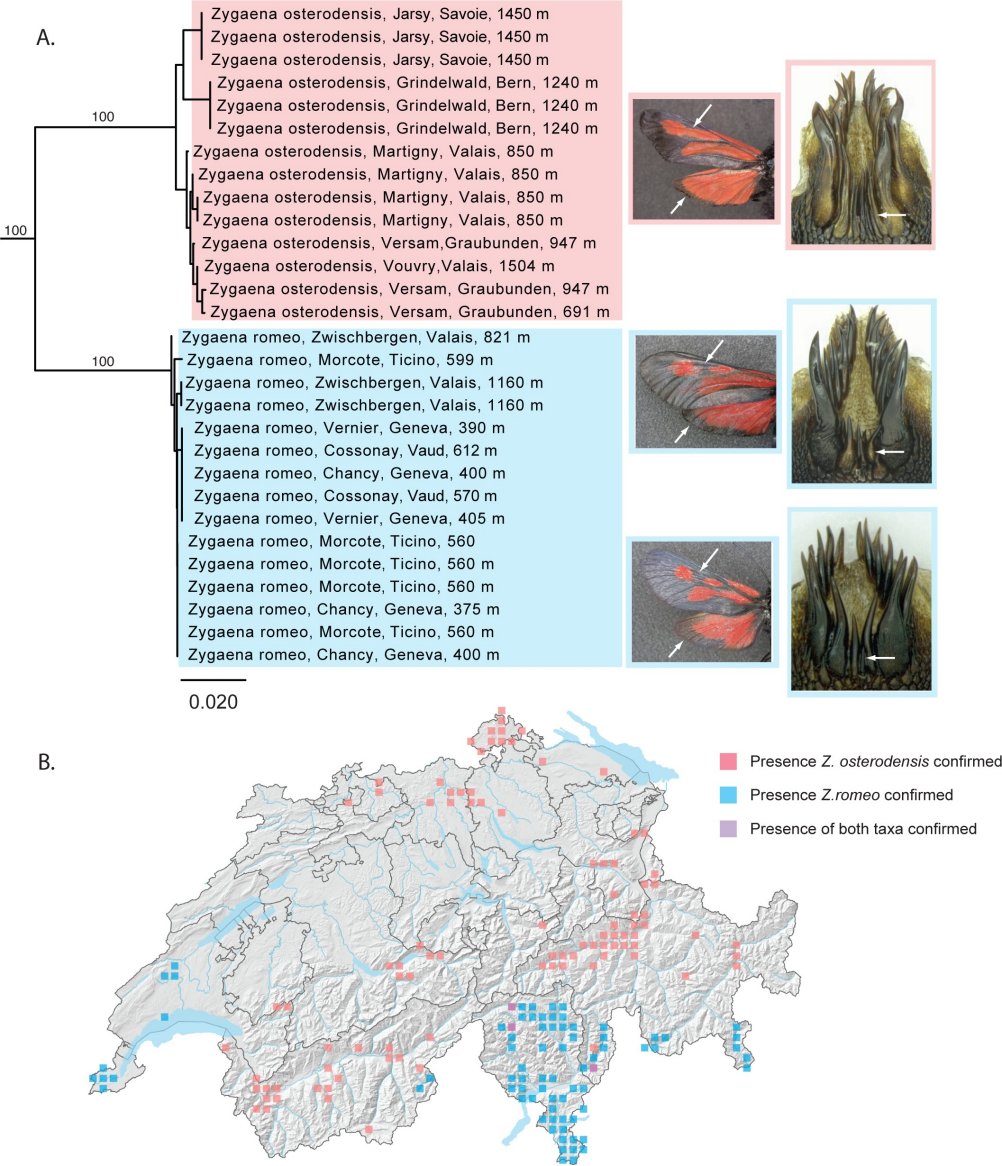
*Zygaena romeo* and *Z*. *osterodensis* form reciprocally monophyletic barcode clusters. (A) NJ tree based on DNA barcodes for *Zygaena romeo* and *Z*. *osterodensis*. Numbers above nodes are NJ bootstrap values over 50% as calculated in PAUP*. Numbers under nodes are maximum likelihood bootstrap values over 50% as calculated in RAxML. White arrows on images in the left column show variation in wing pattern commonly associated with (although not necessarily diagnostic of) these taxa. White arrows on images in right column show variation in the lengths of the spines at the base of the lamina dorsalis, the main criterion used for identification: *Zygaena osterodensis* (upper right) collected in Fully (VS), with relatively long spines; *Z*. *romeo* from Meride (TI) (center right), with relatively short spines; and *Z*. *romeo* from Ferreyres (VD), with spines that are intermediate in length. Images represent morphologically typical populations but do not represent individuals sequenced for this study. (B) Map of Switzerland showing distribution of *Z*. *osterodensis* (pink squares) and *Z*. *romeo* (blue squares). Squares represent 5km^2^ quadrats. Localities where both species are found in the same quadrat are shown as purple squares. Note: Colored squares represent all data present in the national database. Certain populations shown on the map may represent historical populations that no longer exist.

Similarly, the two species of forester moths *Zygaena romeo* and *Z*. *osterodensis* are closely related and often difficult to separate based on morphology [38]. *Zygaena osterodensis* is widely distributed from Spain to Eastern Europe, while *Z*. *romeo* is present in Italy, southern Switzerland and locally in France. The distribution of both species overlaps slightly and a handful localities are known in France and Italy where both species occur syntopically [39]. In most of these localities, the main flight season of *Z*. *romeo* occurs approximately two weeks after that of *Z*. *osterodensis*, with little or no overlap [38]. In Switzerland, it has so far been assumed that *Z*. *romeo* was restricted to the southern flanks of the Alps [40]; all other populations have so far been attributed to *Z*. *osterodensis*. Guenin (2012) [39] documented variation in the genitalia of both males and females of the Z. *osterodensis – Z*. *romeo* complex in Swiss populations south of the Alps. He identified most southern specimens as *Z*. *romeo*, although he reported what appeared to be one female and two males (and two additional specimens with imprecise locality data) of *Z*. *osterodensis*, concluding that both taxa possibly co-occur in Switzerland south of the Alps. In western Switzerland, in a region extending from Geneva to approximately 20 km north of Lausanne, a population (hereafter referred to as the “Geneva population”) occurs that exhibits genitalia that are more like that of *Z*. *romeo* but with some cases of intermediate individuals (Fig 3). The wing pattern of these specimens is more reminiscent of *Z*. *romeo* (Fig 3); the habitus of the larva clearly suggests *Z*. *romeo* and not *Z*. *osterodensis* (not shown). These specimens were initially identified as *Z*. *osterodensis* [39] based on male and female morphology. However, one specimen was identified as *Z*. *nevadensis*, a distinct species not occurring in Switzerland; this specimen has been considered incorrectly labeled [40]. We examined this specimen and, based on the structure of the antennae and genitalia, confirm that it is a male of *Z*. *romeo*, not *Z*. *nevadensis*.

In our molecular analyses, *Z*. *romeo* and *Z*. *osterodensis* formed two well-separated and reciprocally monophyletic clades (Fig 3, minimum genetic distance between species 8.84%). All eight individuals sampled south of the Alps clustered in the *Z*. *romeo* clade, in agreement with both genitalia and wing pattern, and the eleven individuals from other parts of the Swiss Alps clustered in the *Z*. *osterodensis* clade, in agreement with their genitalia (substantial variation is observed in the wing pattern). Specimens from the atypical Geneva population thus far attributed to *Z*. *osterodensis* clustered with *Z*. *romeo*. We also sampled a population in France, some 50 km south of Geneva, morphologically clearly attributed to *Z*. *osterodensis* and clustering with other *Z*. *osterodensis*, indicating that populations of both *Z*. *romeo* and *Z*. *osterodensis* exist in relatively close proximity in the region around Geneva. In summary, our results indicate that *Z*. *romeo* and *Z*. *osterodensis* can be separated using DNA barcodes, but also highlight more variation in the structure of male genitalia than hitherto recognized, possibly testifying to some level of introgression or gene flow. In addition, our results confirm the mosaic-like distribution of *Z*. *osterodensis* and *Z*. *romeo* in Switzerland and neighboring France, with *Z*. *osterodensis* occurring in slightly cooler habitats, and *Z*. *romeo* restricted to thermophilic, low-elevation habitats. Future studies of syntopic populations should closely examine variation in genitalia, as well as individuals exhibiting morphological characters that are intermediate between both taxa.

### Para- and polyphyletic taxa

Species paraphyly may result from genetic phenomena, including events such as incomplete lineage sorting and mitochondrial introgression, as well as methodological phenomena, including identification errors and the implementation of systems of taxonomic classification that do not correspond to the biological reality of species [19,41]. While these phenomena have different underlying causes, all tend to occur when dealing with recently diverged, morphologically similar species. In our analysis of Swiss diurnal Lepidoptera, we recovered nine species pairs or trios that were paraphyletic at the barcode locus.

Four species pairs (eight species) were para- or polyphyletic without sharing barcodes: *Pyrgus malvae* – *P*. *malvoides*, *Adscita statices – A*. *alpina*, *Plebejus argyrognomon* – *P*. *idas* and *Erebia manto – E*. *bubastis*. Paraphyletic or even polyphyletic species may still be correctly identified using the DNA barcode, provided that barcode haplotypes are unique to individual species [13,42]. A 2011 study by Hausmann et al. [15] defined a “diagnostic” barcode as one in which “barcode clusters show constant differences from all other species recognized through classic entomological approaches.”. In other words, diagnostic barcodes are those which are unique to certain species, regardless of whether all individuals of a certain species emerge together in clustering analyses. Under this definition, the haplotypes for the eight taxa mentioned above, being species-specific, allowed for their successful identification and raised the identification success rate of the barcode to 93.5% across Swiss samples. In order to evaluate the robustness of these results to wider geographic sampling, all sequences from these eight species were queried against other available sequences on BOLD. In each case, at least one sequence from each species was identical to a haplotype from its sister species. Assuming identifications of the specimens on BOLD are correct, the ability of the barcode to differentiate members of these species pairs is not robust to sampling outside of Switzerland or perhaps even upon increased sampling within Switzerland. The case of *Erebia manto – E*. *bubastis* is discussed below in the section “Contribution of the barcode to clarifying taxonomic boundaries”.

In the remaining six cases, individuals from closely related species pairs or trios shared barcodes with another member of the same group: *Cupido osiris – C*. *minimus*, *Erebia tyndarus* – *E*. *arvernensis* – *E*. *nivalis*, *Erebia ligea* – *E*. *euryale*, *Coenonympha darwiniana* – *C*. *gardetta*, *Zygaena minos – Z*. *purpuralis* and *Pyrgus alveus* – *P*. *warrenensis* – *P*. *accreta*. In some cases, including the *Erebia tyndarus* complex and the *Pyrgus alveus* complex, an individual of each species in the complex shares a barcode with at least one individual of another species and each species represented by more than one sequence is either para- or polyphyletic. As the *Erebia tyndarus* complex has been discussed in depth elsewhere [43], we will simply conclude that the DNA barcode cannot be used to separate the members of this complex in Switzerland, nor in Europe (S2 Fig), in agreement with the findings of other authors [20,44]. The *Pyrgus alveus* complex, however, merits further discussion here, as do *Coenonympha darwiniana* - *C*. *gardetta* and *Erebia ligea* - *E*. *euryale*.

In Switzerland, the *Pyrgus alveus* complex includes three species: *P*. *alveus*, *P*. *accreta* and *P*. *warrenensis*. The distributions of *Pyrgus alveus* and *P*. *accreta* are considered disjunct, with *P*. *alveus* distributed throughout the Alps and *P*. *accreta* in the Jura. The two taxa are indistinguishable based on wing morphology in both males and females. Some authors differentiate them based on slight differences in the relative proportions of the male genitalia [45] but others have noted that while these differences may be minimal within populations, they are far more variable among populations, even within the same species [40], calling into question their utility for differentiating these two taxa. Given their supposedly non-overlapping geographical distributions, locality is often used as a criterion for identification. The status of *P*. *accreta* varies depending on the author, with some considering it a distinct species [40], some considering it a subspecies of *P*. *alveus* [46] and others considering it a synonym of *P*. *alveus* [47]. Like *P*. *alveus*, *P*. *warrenensis* is also found throughout the Alps but in much more restricted areas in the cantons of Valais, Bern and Graubunden principally at altitudes between 2000m and 2650m. *Pyrgus warrenensis* is usually smaller and darker than *P*. *accreta* and *P*. *alveus* and also differs with regard to characters in male and female genitalia [40,48].

In our NJ analyses, both *Pyrgus alveus* and *P*. *warrenensis* emerge as paraphyletic with respect to other members of the complex in a mixed cluster together with *P*. *accreta* (the latter represented by a single specimen in our analyses). *Pyrgus alveus* and *P*. *warrenensis* are both characterized by greater intraspecific divergence than distance to a nearest-neighbor (*P*. *alveus* max. intraspecific divergence = 0.49%, minimum distance to nearest neighbor, *P*. *accreta*, = 0.0%; *P*. *warrenensis* max. intraspecific divergence = 0.62%, minimum distance to a nearest neighbor, *P*. *accreta* = 0.0%). While the intraspecific divergence of *P*. *accreta* could not be evaluated, it also shares a barcode with its nearest neighbor, *P*. *warrenensis*. Two groups of individuals shared haplotypes. The first group consisted of two individuals of *P*. *alveus* from Piz Beverin and Fusio (cantons of Graubunden and Ticino, respectively), *P*. *accreta* from the Col du Marchairuz (canton of Vaud) and *P*. *warrenensis* from the Kiental (canton of Bern). The second group exhibiting identical haplotypes included a specimen of *P*. *alveus* from Val de Gallo (canton of Graubunden) and a specimen of *P*. *warrenensis* from Piz Sesvenna (canton of Graubunden).

The DNA barcode is thus unable to unambiguously distinguish the three members of the *P*. *alveus* complex in Switzerland. Huemer and Wiesmair (2017) [20] report similar findings for Austrian members of the complex, finding no barcoding gap between *P*. *alveus* and *P*. *warrenensis*. A study based on a combination of mitochondrial genomes and nuclear ribosomal DNA also reported paraphyly in the *P*. *alveus* complex (including six taxa in total from throughout the Palearctic region), without specifying the nature of the paraphyly [49]. An analysis of Swiss barcodes together with other European sequences has been included in the Supporting Information (S3 Fig).

The presence of shared haplotypes between individuals of *Pyrgus alveus* from the Alps and a single individual of *P*. *accreta* from the southwestern Jura Mountains may be explained by recent introgression or incomplete lineage sorting. The morphological, genetic and ecological similarities between these taxa, however, also allow for the possibility that populations at the southwestern extreme of the Jura Mountains, once thought to be *P*. *accreta*, may in fact be *P*. *alveus*. Further sampling is required, namely from populations at the northeastern end of the Jura Mountains, as well as from neighboring regions including southern Germany and western France, to further clarify the status of the *P*. *alveus* complex In the Jura Mountains and of *P*. *accreta* in Switzerland. Pending more detailed studies, we retain this taxon on the Swiss national species list, as has been done in Germany [26].

In contrast, *Pyrgus alveus* and *P*. *warrenensis* may be differentiated based on a series of criteria, including consistent differences in both male and female genitalia, the small size and reduced white wing markings of *P*. *warrenensis* and, to some degree, the high-altitude distribution of *P*. *warrenensis*. The two taxa overlap for a portion of their distributions, namely between 2000–2400m, and have been observed in flight together on the Täschalp (canton of Valais), providing evidence that they remain morphologically distinct even in syntopy [36,50]. Yet the presence of shared barcodes suggests either introgression or incomplete lineage sorting. A more complete dataset including nuclear markers (such as SNPs) will be needed to address this question more appropriately. For the moment, we will continue to recognize *P*. *warrenensis* as a separate species from *P*. *alveus* in Switzerland based on consistent differences in both morphology and habitat preference.

In Switzerland, *Coenonympha gardetta* occurs throughout the Alps typically between altitudes of 1400m to 2500m and above. In the region of the Simplon in the eastern Valais, in the canton of Ticino and in the southern valleys of the canton of Graubunden, however, *C*. *gardetta* is replaced to some degree by *C*. *darwiniana*. At zones of contact between the two species, individuals that are morphologically intermediate between *C*. *gardetta* and *C*. *darwiniana* are known to occur. In our analyses of Swiss sequences, a single individual identified by several specialists as *Coenonympha darwiniana* shared a barcode with individuals of *C*. *gardetta*. This individual of *C*. *darwiniana* was collected from Obergoms (canton of Valais), a locality where purportedly intermediate individuals have been observed in the past [36]. *Coenonympha darwiniana* has been proposed as a stabilized hybrid between *C*. *gardetta* and *C*. *arcania* [51] and the presence of shared mitochondrial haplotypes in *C*. *darwiniana* and *C*. *gardetta* suggests either that hybridization between *C*. *darwiniana* and *C*. *gardetta* was still possible in the recent past (and/or that it may still be a reality), that mitochondrial haplotypes of these species are still in the process of segregating, or both. Despite possible introgression, we continue to treat both taxa as distinct biological species due to consistent differences in the morphology and altitudinal distribution between these two taxa, as well as the emergence of the majority of Swiss specimens into species-specific barcode clusters. We find no evidence of introgression between either *C*. *darwiniana* or *C*. *gardetta* and *C*. *arcania*.

*Erebia ligea* and *E*. *euryale* are considered closely related yet distinct species that are generally distinguishable based on both wing pattern and male and female genitalia; to the best of our knowledge, hybrids between the two are unreported in Switzerland. Both taxa are found in the Jura and throughout the Alps and are absent across a wide swath of the Plateau. While *Erebia ligea* is distributed from low altitude valleys up through the subalpine zone in Switzerland, *E*. *euryale* is most abundant from approximately 800m through the subalpine zone. In our analyses, one individual of *E*. *ligea* from Safien (canton of Graubunden) shared a barcode with specimens of *E*. *euryale*. Our results suggest that introgression as a result of hybridization between *E*. *ligea* and *E*. *euryale* may occur along zones of contact or that mitochondrial haplotypes of these species are still in the process of segregating in certain populations. Paraphyly within the species pair *E*. *ligea* - *E*. *euryale* was also reported for Romanian Lepidoptera [13].

### Potential cases of cryptic diversity

Cryptic diversity, defined as the presence of two or more morphologically similar species within a group previously classified as a single species [52], is a well-known phenomenon in Lepidoptera [14,16,18]. Some studies have proposed the identification of a standard genetic threshold for establishing taxonomic boundaries, namely because it would assign an unambiguous quantitative value to the subjective concept of “deep” genetic divergences [53]. Levels of intraspecific genetic diversity vary greatly from one taxonomic group to another, however, and the use of a simple genetic threshold for species delimitation without a sound understanding of underlying species biology and ecology may lead to spurious taxonomy [54,55,56]. Furthermore, it is not possible to distinguish the presence of cryptic lineages from high levels of intraspecific divergence based on a numerical threshold alone. It is, however, useful to designate a value for the purpose of discussion, as well as to highlight the presence of genetic lineages that may be of conservation importance.

A study of temperate Canadian Lepidoptera reported that divergence values between species were typically greater than 3% and that at this threshold, barcode-based identifications recovered the same species as morphology-based identification with a success rate of 98% [57]. Other studies have used a threshold of 2% in order to highlight genetically diverse groups that merit further attention [13]. In this study, eleven species showed levels of intraspecific divergence over 2%: *Zygaena lonicerae*, *Limenitis camilla*, *Aphantopus hyperantus*, *Zygaena transalpina*, *Plebejus argyrognomon*, *Thymelicus lineola*, *Erebia manto*, *Thymelicus sylvestris*, *Eumedonia eumedon*, *Melitaea athalia* (also including *M*. *nevadensis*), and *Zygaena filipendulae*.

*Melitaea athalia* and *M*. *nevadensis* exhibit a parapatric distribution in Switzerland, with *M*. *athalia* known from the Jura and the northern regions of the Plateau and *M*. *nevadensis* found throughout the northern flank, central and southern flank of the Alps. Adults can be differentiated solely on the basis of structural differences in the male genitalia; morphological differences between these taxa have also been reported in eggs, full grown larvae and pupae [58]. To the best of our knowledge, no morphological criteria exist to differentiate females. In Switzerland, a zone of contact exists along an axis that runs from the southeast to the northwest of the country in which the males of certain populations exhibit genitalia intermediate between both taxa [36].

**Fig 4:**
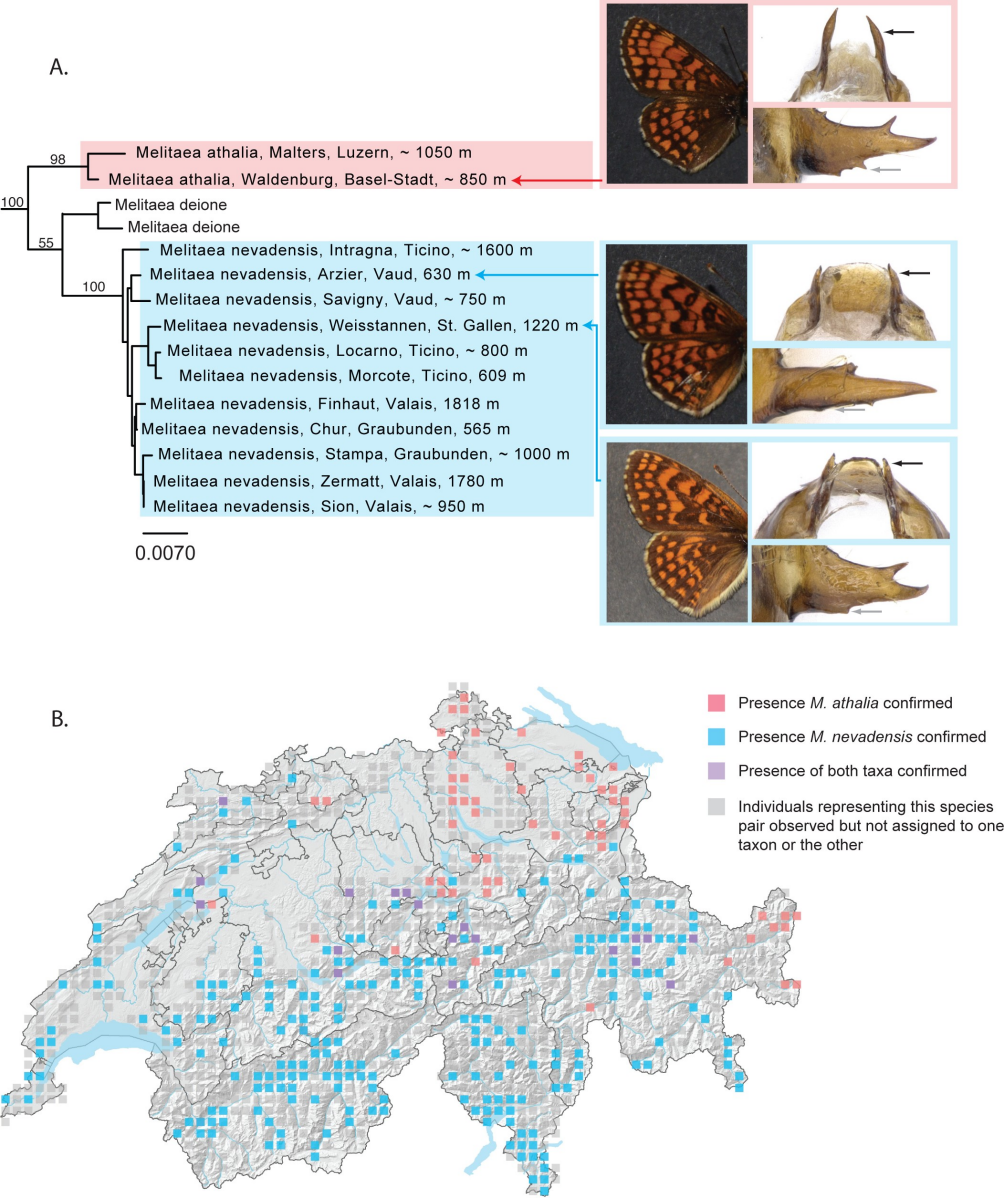
*Melitaea athalia* and *M*. *nevadensis* as independently evolving mitochondrial lineages. (A). NJ tree based on DNA barcodes for *Melitaea athalia* and *M*. *nevadensis*. Numbers above nodes are NJ bootstrap values over 50% as calculated in PAUP*. Numbers under nodes are maximum likelihood bootstrap values over 50% as calculated in RAxML. Images in left column show the dorsal habitus of these taxa (non-diagnostic). Black arrows on the images in the right column indicate the length of the uncus and grey arrows the base of the genital valve for each of three taxa: *Melitaea athalia* (upper right), with uncus relatively long and genital valve with multiple teeth at the base; a typical *Melitaea nevadensis* (middle right), with uncus relatively short and genital valve untoothed at the base; and a morphologically atypical specimen of *Melitaea nevadensis* (lower right) with the uncus relatively short but exhibiting a genital valve with a pronounced tooth at the base. (B) Map of Switzerland showing distribution of *M*. *athalia* (pink squares) and *M*. *nevadensis* (blue squares). Squares represent 5km^2^ quadrats. Localities where both species are found in the same quadrat are shown as purple squares. Localities representing individuals for which genitalia have not been examined and which have thus not been assigned to one taxon or the other are shown as grey squares. Note: Colored squares represent all data present in the national database. Certain populations shown on the map may represent historical populations that no longer exist.

Long considered as a subspecies of *Melitaea athalia*, *M*. *nevadensis* was elevated to species rank when an analysis of a three-gene dataset (CO1, wingless and EF-1 alpha) demonstrated that *M*. *athalia* was not the sister taxon to *M*. *nevadensis* as previously thought but was more closely related to *M*. *deione*, *M*. *britomartis*, *M*. *ambigua* and *M*. *caucasogenita* [59]. In our analyses of thirteen Swiss specimens, *M*. *athalia* and *M*. *nevadensis* emerge as two independent monophyletic mitochondrial lineages, separated by a minimum genetic distance of 4.14% (Fig 4). Furthermore, individuals of *Melitaea nevadensis* are more closely related to *M*. *deione* than to *M*. *athalia*.

The deep mitochondrial divergence seen between these taxa and the apparent absence of mitochondrial introgression among Swiss samples allows for the possibility that these lineages have long evolved independently of one another. The presence of ostensibly hybrid populations along the zone of contact, however, implies that populations have not yet reached reproductive isolation [59]. An analysis of Swiss sequences together with sequences from other European populations show that *Melitaea nevadensis* is more similar to both *M*. *britomartis* and *M*. *deione* than to *Melitaea athalia* at the barcode locus. The labeling of many individuals simply as “*Melitaea athalia*” on the BOLD platform, even from localities typically associated with *M*. *nevadensis*, makes it difficult to discern whether there is evidence of mitochondrial introgression among European samples of *M*. *athalia* and *M*. *nevadensis* (S4 Fig). Huemer and Wiesmair [20] report that certain Austrian individuals exhibiting genitalia consistent with *M*. *athalia* emerge in a barcode cluster with individuals of *M*. *nevadensis* (as *M*. *celadussa*). *Melitaea nevadensis* and *M*. *athalia* last shared a common ancestor approximately seven million years ago [56] and they may have evolved in allopatry throughout much of this time, only more recently coming into contact in a narrow region subtending sections of Switzerland and its neighboring countries, with some degree of hybridization still possible in this area. According to Descimon and Mallet (2009) [60], approximately 16% of European butterfly species are capable of hybridizing with another species in the wild and 8% are also capable of producing viable offspring, suggesting that the case of *Melitaea athalia – M*. *nevadensis* is not unique.

Haldane’s Rule predicts that in a cross between two species, the heterogametic sex is the one most likely to be rare, absent or inviable. As applied to Lepidoptera, maternally inherited mitochondrial markers would be less likely to be transmitted from one species to another than nuclear markers. Patterns seen in *M*. *athalia* and *M*. *nevadensis*, namely the marked divergence between mitochondrial lineages combined with the apparent capacity of these two taxa to interbreed, are consistent with the outcome expected if Haldane’s Rule were true for these populations. An exploration of multiple populations using a combination of nuclear and mitochondrial markers, especially along the zone of contact, will reveal whether nuclear markers follow the same patterns as mitochondrial or whether gene flow between these two taxa may be ongoing. Further characterization of the taxonomic status of *M*. *athalia* and *M*. *nevadensis* should depend, at least in part, on the extent of the zone of hybridization and whether it represents a long, gradual cline or whether the transition from one taxon to the other is abrupt and contains few intermediate forms. Currently registered in the national database as subspecies, *M*. *athalia* and *M*. *nevadensis* will be considered as distinct species in Switzerland in the future, as per Leneveu et al. (2009) [59].

For both *Thymelicus lineola* (maximum intraspecific divergence = 3.27%) and *Zygaena filipendulae* (maximum intraspecific divergence = 5.63%), Switzerland represents an important zone of contact between mitochondrial haplotypes in Europe and the genetic structure observed within each of these taxa is associated with geographic distribution. To the best of our knowledge, mitochondrial divergence in *T*. *lineola* is not accompanied by discernible morphological or behavioral differences in Switzerland or elsewhere. In our analyses, specimens from Mesocco, Chur and Stampa (canton of Graubunden), as well as from Derborence (canton of Valais) are reciprocally monophyletic from a cluster consisting of specimens from Tramelan (canton of Bern), the Grand Combin (canton of Valais) and the Col du Pillon (canton of Vaud). When analyzed together with other European sequences on BOLD, five clusters emerge, one corresponding to a single specimen from Turkey; a second corresponding to two individuals, one from Spain and one from Germany; the third and fourth corresponding exclusively to haplotypes from European populations and a fifth to specimens sampled from both Europe and North America (S5 Fig). A zone of contact, which runs through Switzerland, exists between two principal European haplotypes. Genetic structure has previously been reported in this taxon [18].

Similarly, *Zygaena filipendulae* is represented by two major clusters in Switzerland, one corresponding to populations from north of the Alps and the other to populations from south of the Alps: specimens collected in La Neuveville (canton of Bern), Waldenburg (canton of Basel-Stadt), Chur (canton of Graubunden), Piz Beverin (canton of Graubunden), Col du Pillon (canton of Vaud), Savigny (canton of Vaud), Visp (canton of Valais) and Chancy (canton of Geneva) clustered independently from a single specimen collected in Morcote (canton of Ticino). When these sequences are analyzed together with other European sequences on BOLD, haplotypes continue to segregate into two major clusters corresponding to northern (Switzerland, Austria, Germany, Finland, Norway, Ukraine and the UK) and southern populations (Italy and southern Switzerland) (S6 Fig). Again, Switzerland lies along the zone of contact between these haplotypes. In contrast to *Thymelicus lineola*, populations of *Z*. *filipendulae* north and south of the Alps exhibit morphological differences. Some authors considered certain populations south of the Alps as belonging to a distinct species *Zygaena stoechadis* (Borkhausen, 1793) [61,62]. More recently, these populations have been considered as a distinct subspecies *Zygaena filipendulae stoechadis* (Borkhausen, 1793) [63]. Further sampling and analyses will help clarify the status of *Z*. *filipendulae*.

The elevated intraspecific divergence seen in *Eumedonia eumedon* (4.43%) is caused by a single specimen collected in Lauenen (canton of Bern); if this specimen is removed, intraspecific divergence for this species drops to 0.46% in Swiss samples. When our sequences are analyzed with 99 other sequences available for this species on BOLD, this individual emerges as the sister taxon to all others and is the only member of its BIN. The identification of the specimen in question was confirmed by multiple experts, the sequences in both directions are clean, no stop codons were detected and a query search using both the contig, as well as the “forward” and “reverse” sequences alone are matched unambiguously to *E*. *eumedon*. Although the possibility exists that this specimen represents a new unique haplotype, another explanation is that this sequence may represent a nuclear copy of a mitochondrial gene.

### Contribution of the barcode to clarifying taxonomic boundaries

For certain species, two distinct subspecies are recognized in Switzerland on the basis of differences in morphology, habitat preference and/or geographical distribution, including *Erebia pronoe vergy – E*. *p*. *psathura*, *Lycaena tityrus tityrus* – *L*. *t*. *subalpina* and *Euphydryas aurinia aurinia* – *E*. *a*. *glaciegenita*. Another case, that of *Erebia manto* – *E*. *bubastis*, involves two closely related taxa whose taxonomic status requires attention. In analyzing the barcodes for these taxa, one of our objectives was to determine whether genetic results provided support in favor of current taxonomic assignments. Each of these cases is outlined below and the implications of our results on future consideration of these taxa in Switzerland is discussed.

#### Erebia pronoe vergy – E. p. psathura

Two subspecies of *Erebia pronoe* are recognized in Switzerland [64]. *Erebia pronoe vergy* is found throughout the northern flank of the Alps, from the canton of Vaud in the southeast to St. Gallen in the northwest, as well from isolated populations in the western Swiss Jura. *Erebia pronoe psathura* is known very locally from the eastern Valais and western Ticino. These two subspecies may be distinguished principally by the shape of the apical margin of the discal band of the third cell on the lower surface of the hindwing. From an ecological perspective, *E*. *p*. *vergy* is associated with limestone substrates, while *E*. *p*. *psathura* is found on siliceous rock, differences likely associated with alternative colonization histories.

**Fig 5:**
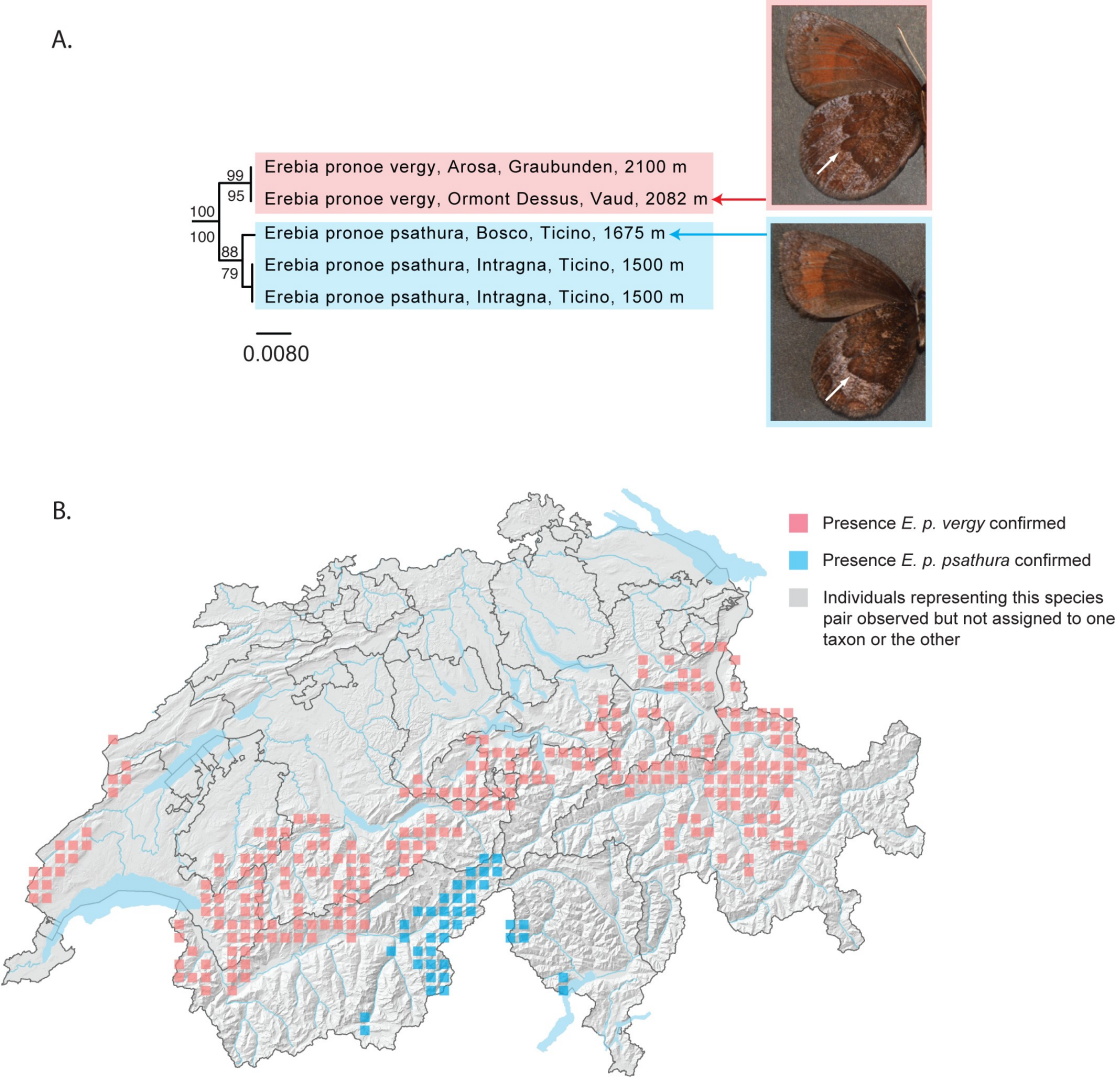
*Erebia pronoe vergy* and *E*. *p*. *psathura* as independently evolving mitochondrial lineages. (A) Neighbor joining (NJ) tree based on DNA barcodes for *Erebia pronoe vergy* and *E*. *p*. *psathura*. Values above nodes are NJ bootstrap values over 50% as calculated in PAUP*. Values under nodes are maximum likelihood bootstrap values over 50% as calculated in RAxML. White arrows indicate the difference in wing pattern used to distinguish these taxa: *E*. *p*. *vergy* (upper image), with the apical margin of the discal band of the third cell strongly convex; and *E*. *p*. *psathura* (lower image), with the apical margin of the discal band of the third cell straight. (B) Map of Switzerland showing distribution of *E*. *p*. *vergy* (pink squares) and *E*. *p*. *psathura* (blue squares), based principally on Sonderegger (2005), as well as on additional data. Squares represent 5km^2^ quadrats. A single quadrat where populations have been observed but not assigned to one taxon or the other is shown as a grey square. Note: Colored squares represent all data present in the national database. Certain populations shown on the map may represent historical populations that no longer exist.

In our analyses, Swiss individuals from populations of *E*. *p*. *vergy* and *E*. *p*. *psathura* are reciprocally monophyletic, corresponding to two independently evolving mitochondrial lineages (Fig 5). We use the relatively elevated genetic distance between these taxa (minimum distance = 1.14%, maximum distance = 1.71%), the allopatric nature of their distributions and the morphological differences between them as evidence supporting the sub-specific status of these taxa. While the differences between them could also be used to support species-level status for *E*. *p*. *vergy* and *E*. *p*. *psathura*, an analysis of Swiss specimens together with individuals from other European populations demonstrates that such a promotion would have taxonomic implications that would go well beyond the scope of this paper (S7 Fig).

#### Lycaena tityrus tityrus – L. t. subalpina

Two subspecies of *Lycaena tityrus* are known in Switzerland. *Lycaena tityrus tityrus* is known from low to middle altitudes. Females of this taxon are characterized by bright orange upper wing surfaces and brightly colored ventral wing surfaces [36]. *Lycaena t*. *subalpina* is known from about 1000 m until 2500 m. Females lack the strong orange wing coloration seen in *L*. *t*. *tityrus* and males are darker [36]. A zone of contact between the two taxa exists at intermediate altitudes throughout at least part of their distributions [60,65].

Descimon (1980) [65] referred to these taxa as “quasi” species and Descimon and Mallet (2009) [60] as “bad” species, namely because interactions between populations are difficult to characterize, particularly at zones of contact. Purportedly hybrid individuals from unspecified localities have been reported by some authors [66]; other authors have reported stabilized hybrid populations in the Bernese Oberland and the Dolomites [67]. First-generation hybrids (*L*. *t*. *tityrus* x *L*. *t*. *subalpina*) from French populations reared under experimental conditions, however, appear to be non-viable [65].

**Fig 6:**
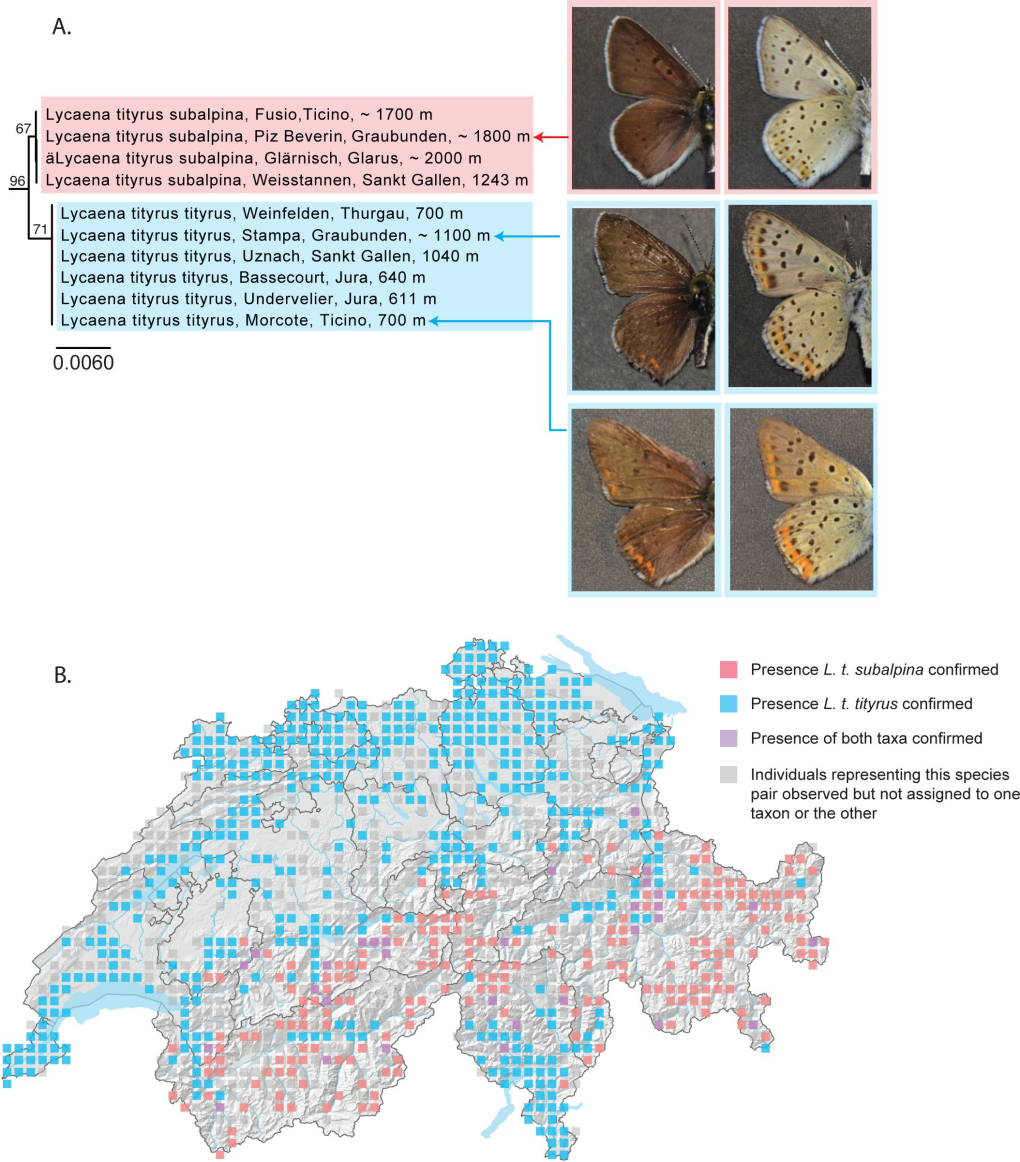
*Lycaena tityrus tityrus* and *L*. *t*. *subalpina* form reciprocally monophyletic barcode clusters. (A) NJ tree based on DNA barcodes for *Lycaena tityrus tityrus* and *L*. *t*. *subalpina*. Values above nodes are NJ bootstrap values over 50% as calculated in PAUP*. Values under nodes are maximum likelihood bootstrap values over 50% as calculated in RAxML Images in left column show the dorsal habitus typically associated with males of these taxa and images in the right column the ventral habitus: *L*. *t*. *subalpina* (upper left and right), lacking orange markings on the upper surfaces and with limited orange markings on the lower surfaces of both fore- and hindwings, and *L*. *t*. *tityrus* (lower left and right), with distinct orange markings on upper and lower surfaces of fore- and hindwings. Certain individuals of *L*. *t*. *tityrus*, clustering with other *L*. *t*. *tityrus*, exhibit wing patterns that are intermediate between the two taxa. (B) Map of Switzerland showing distribution of *L*. *t*. *subalpina* (pink squares) and *L*. *t*. *tityrus* (blue squares). Squares represent 5km^2^ quadrats. Localities where both subspecies are found in the same quadrat are shown as purple squares. Localities where individuals have been observed but not assigned to one taxon or the other are shown as grey squares. Note: Colored squares represent all data present in the national database. Certain populations shown on the map may represent historical populations that no longer exist.

Our analyses show that populations of *L*. *t*. *subalpina* from the Alps and populations of *L*. *t*. *tityrus* from the lowlands represent two independently evolving mitochondrial lineages in Switzerland, separated by a minimum genetic distance of 0.33% (Fig 6). This genetic proximity, in combination with what appears to be at least some degree of reproductive incompatibility, suggests that these may be young, recently diverged taxa still in the process of establishing reproductive isolation. An analysis of sequences from other European populations also recovers two major reciprocally monophyletic clusters representing *L*. *t*. *tityrus* and *L*. *t*. *subalpina* (S8 Fig). For the moment we retain these taxa as subspecies of *L*. *tityrus*. An improved understanding of the dynamics between populations of these two taxa will require a larger dataset, namely one including nuclear genes. Huemer and Wiesmair (2017) [20] also reported reciprocally monophyletic clades corresponding to *L*. *t*. *tityrus* and *L*. *t*. *subalpina* for Austrian populations.

#### Euphydryas aurinia aurinia – E. a. glaciegenita

In Switzerland, two subspecies of *Euphydryas aurinia* are recognized. *Euphydryas aurinia aurinia* is found mostly in association with wetlands (although sometimes also with drylands) at low to mid-elevation, occurring up until 1500m. At higher altitudes (1800-2600m), it is replaced by the subspecies *Euphydryas aurinia glaciegenita*, smaller, darker and exhibiting a preference for alpine grasslands. Between 1200 and 1800 m, for example in the Prealps in the canton of Vaud, forms are known that are morphologically intermediate between the two and rearings have demonstrated that these two taxa are able to hybridize and produce viable offspring [36].

**Fig 7:**
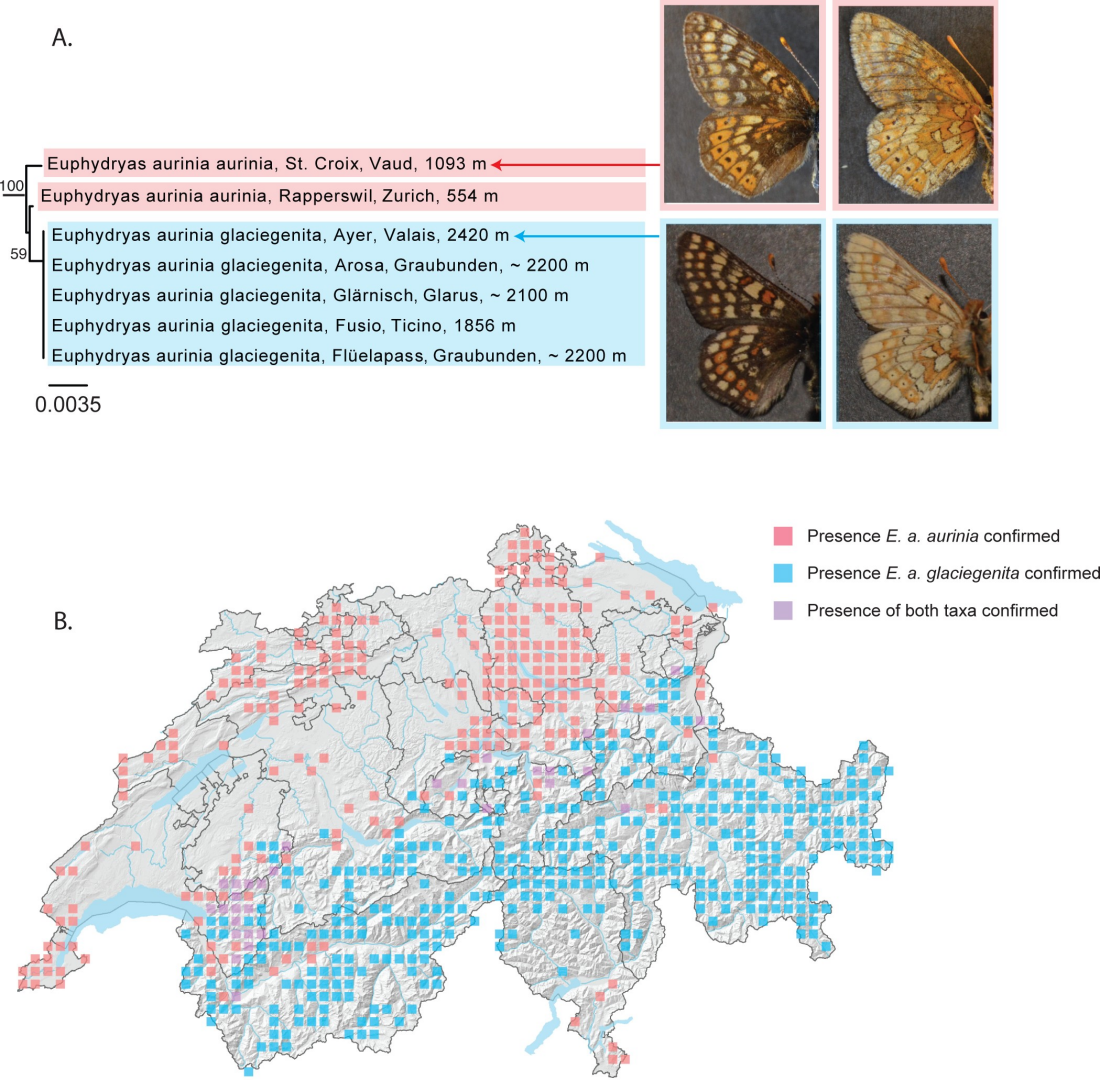
*Euphydryas aurinia glaciegenita* emerges from within a paraphyletic *Euphydryas aurinia aurinia*. (A) NJ tree based on DNA barcodes for *Euphydryas aurinia aurinia* and *E*. *a*. *glaciegenita*. Numbers above nodes are NJ bootstrap values over 50% as calculated in PAUP*. Numbers under nodes are maximum likelihood bootstrap values over 50% as calculated in RAxML. Images in left column show the dorsal habitus typically associated with these taxa and images in the right column the ventral habitus: *E*. *a*. *aurinia* (upper left and right), brighter than *E*. *a*. *glaciegenita* and with black ocelli highly visible toward the posterior margin on both upper and lower surfaces of the hindwings, and *E*. *a*. *glaciegenita* (lower left and right), darker and with weak black ocelli toward the posterior margin on both upper and lower surfaces of the hindwings. (B) Map of Switzerland showing distribution of *E*. *a*. *aurinia* (pink squares) and *E*. *a*. *glaciegenita* (blue squares). Squares represent 5km^2^ quadrats. Localities where both subspecies are found in the same quadrat are shown as purple squares. Note: Colored squares represent all data present in the national database. Certain populations shown on the map may represent historical populations that no longer exist.

An analysis of our sequences demonstrates that *Euphydryas aurinia glaciegenita* emerges from within a paraphyletic *Euphydryas aurinia aurinia* (Fig 7). No haplotypes are shared and the barcode is successful at differentiating these taxa. While they are not reciprocally monophyletic, differences in morphology and habitat preference are associated with genetic differences. We will continue to recognize two subspecies in Switzerland, particularly important given that these taxa represent different conservation concerns according to IUCN criteria: *E*. *a*. *aurinia* is considered “Endangered” in Switzerland, while *E*. *a*. *glaciegenita* is considered “Least concern”.

#### Erebia manto – E. bubastis

In Switzerland, *Erebia manto* and *E*. *bubastis* are considered closely related taxa due to similarities in their morphology and ecology. *E*. *manto* is common throughout the northern foothills of the Alps and occurs more sporadically in the southern foothills, while *E*. *bubastis* is found in restricted localities scattered throughout the cantons of Valais, Ticino and Graubunden. Currently recognized at the species-level, they were previously recognized as subspecies in Switzerland, with populations of *E*. *manto* assigned to the subspecies *Erebia manto mantoides* and *E*. *bubastis* to the subspecies *Erebia manto bubastis* [64]. The two taxa can be differentiated based on distinct differences in wing pattern and male genitalia. Thus far they have not been reported from the same localities.

**Fig 8:**
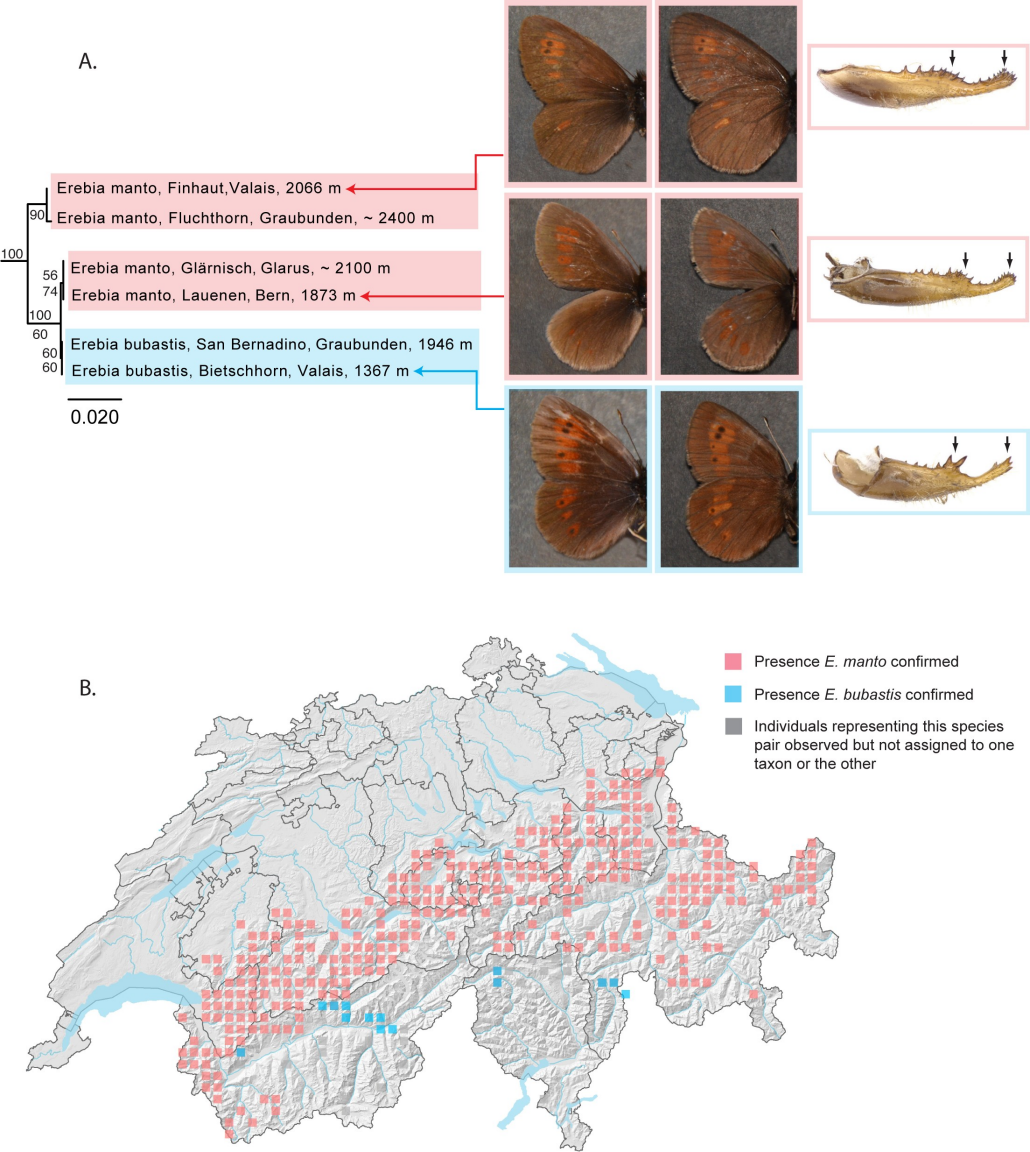
*Erebia manto* paraphyletic with respect to *E*. *bubastis* based on DNA barcodes. (A) Neighbor joining (NJ) tree based on DNA barcodes for *Erebia manto* and *E*. *bubastis*. Numbers above nodes are NJ bootstrap values over 50% as calculated in PAUP*. Numbers under nodes are maximum likelihood bootstrap values over 50% as calculated in RAxML. Images in left and center columns show the dorsal and ventral habitus typically associated with (although not necessarily diagnostic of) these taxa. Images in the right column show the genital valve for each of three taxa: *E*. *manto*, with both the swelling and the tip of the genital valve with numerous small teeth (upper and middle right) and *E*. *bubastis*, with both the swelling and the tip of the genital valve with few, relatively large teeth. (B) Map of Switzerland showing distribution of *E*. *manto* (pink squares) and *E*. *bubastis* (blue squares). Squares represent 5km^2^ quadrats. Localities where populations have been observed but not assigned to one taxon or the other are shown as grey squares. Note: Colored squares represent all data present in the national database. Certain populations shown on the map may represent historical populations that no longer exist.

Our dataset included four specimens of *Erebia manto* and two of *Erebia bubastis* representing localities throughout the Swiss Alps. Neighbor-joining analyses recovered two major clusters separated by a minimum genetic distance of 2.15%. The first cluster includes two specimens of *Erebia manto* from Plan de Marais (canton of Valais) and Scuol (canton of Graubunden). The second cluster includes two specimens of *E*. *manto* from Lauenen (canton of Bern) and Luchsingen (canton of Glarus) and two specimens of *E*. *bubastis* from Piz Pian Grande (canton of Graubunden) and Bietschhorn (canton of Valais). Within this second cluster, both specimens of *E*. *manto* cluster together, as do both specimens of *E*. *bubastis*, the two lineages separated by a minimum genetic distance of 0.15% (Fig 8).

*Erebia bubastis* thus emerges as an independently evolving mitochondrial lineage from within *E*. *manto*. Male genitalia are markedly different between these taxa, far more so than between other sibling taxa from the genus *Erebia* widely regarded as individual species, e.g. *Erebia sudetica* and *E*. *melampus*. Cases of morphologically distinct species exhibiting low genetic divergences have been reported for other insects [68] and despite the low genetic distance between *E*. *bubastis* and *E*. *manto*, the striking differences in the structure of the male genitalia, and the precopulatory barrier to reproduction that these differences are likely to represent, lead us to continue to recognize two distinct species in Switzerland.

The consideration of *E*. *bubastis* as a distinct species from *E*. *manto* renders *E*. *manto* paraphyletic at the barcode locus. Both body size and wing pattern vary considerably among individuals of *E*. *manto*, despite the homogeneity of male genitalia. For the moment, we find no association between the relatively elevated genetic distance among certain individuals of *Erebia manto* and any particular morphs of this taxon, nor for that matter, with any other behavioral or geographical attribute. Further studies of these taxa are in order, namely to better understand the relationship between *E*. *manto* and *E*. *bubastis*, as well as relationships within *E*. *manto*.

### Endemism

The Alps are a major biodiversity hotspot and represent a conservation priority for Switzerland. While there are no diurnal Lepidoptera that are strictly endemic to Switzerland (i.e. whose distributions fall entirely within Swiss borders), three subalpine or alpine species are considered “partially endemic” by the Swiss Federal Office for the Environment. This status is reserved for those species whose distributions are limited to restricted zones of Switzerland and neighboring countries and whose total area of distribution is less than 10,000 km^2^ [69]. To the best of our knowledge, we present the first published barcode sequences for *Erebia christi*, a subalpine species restricted to small populations in the region of the Simplon and the adjacent region in Italy, as well as the first Swiss barcode for *Kretania trappi*, a species found only in the canton of Valais [36] and in the Italian Alps [70]. We also present the first Swiss barcode for *Erebia flavofasciata*, an alpine species known from populations scattered throughout the cantons of Graubunden and Ticino, as well as from the Italian and Austrian Alps.

A number of infraspecific taxa are tentatively considered endemic to Switzerland, including *Melitaea deione berisalii* Rühl, 1891 and Swiss populations of *Kretania trappi* (Verity, 1927) (previously referred to as *Plebejus pylaon trappi* (Verity, 1927)). In order to explore whether DNA barcode sequences provide evidence in support of the “endemic” status of these taxa, we analyzed our sequences with all other available sequences on the BOLD platform. Our results provide a genetic perspective on the status of each of these taxa based on their relationships to other populations across Europe.

*Melitaea deione* is distributed throughout Europe from Portugal east to southern France, southern Switzerland and northern Italy, as well as in Morocco and Algeria [58]. The Swiss subspecies, *M*. *d*. *berisalii*, is found only in the Valais. *Kretania trappi* is sparsely distributed in the Swiss and Italian Alps. Swiss populations of *K*. *trappi*, considered morphologically distinct from those found in Italy, are restricted to limited localities in the Valais, while Italian populations are found only in the Aosta Valley and Trentino-Alto Adige. In analyses of DNA barcodes, two individuals of *M*. *d*. *berisalii* emerge in a monophyletic cluster exhibiting a minimum genetic distance of 0.64% from their nearest neighbor, an individual from the French Alps (S9 Fig). A single individual of *K*. *trappi* exhibits a minimum genetic distance of 0.16% from its nearest neighbor, a specimen from Trentino-Alto Adige (S10 Fig). These preliminary results demonstrate that Swiss populations of *M*. *d*. *berisalii* exhibit unique haplotypes among sequenced individuals and support the status of this taxon as endemic to Switzerland. The relatively low genetic distance between a Swiss individual of *K*. *trappi* and Italian populations of closely related taxa suggest that the endemic status of *K*. *trappi* in Switzerland should be explored further. More comprehensive sampling will help to better characterize the status of both *M*. *d*. *berisalii* and *K*. *trappi* in Switzerland.

## Conclusion

We present the first DNA barcode library for the diurnal Lepidoptera of Switzerland, representing 96.9% of the resident fauna and allowing for the unambiguous identification of nearly 90% of the species sampled. The remaining 10% represent pairs or trios of closely related taxa exhibiting higher levels of intraspecific divergence than distance to a nearest-neighbor. While some of these, e.g. *Erebia ligea – E*. *euryale*, appear to represent largely morphologically and genetically distinct taxa undergoing some degree of either introgression or incomplete lineage sorting, others, e.g. *Pyrgus alveus* – *P*. *accreta* – *P*. *warrenensis*, represent groups whose taxonomic status requires further examination. An integrated approach using a combination of DNA barcoding and morphology-based taxonomy clearly provides the highest success rate for species identification in Switzerland (Table 1), over 98% for males and over 96% in females. When used in tandem with morphological methods, the barcode thus provides a diagnostic edge over traditional morphology-based taxonomy alone.

The DNA barcode has great potential as a tool for the enhancement of conservation strategies in Switzerland. It shows promise for improving the resolution of biodiversity surveys by providing a means of differentiating otherwise indistinguishable taxa, as in the case of female *Hipparchia fagi* and *H*. *genava*, and by providing a criterion beyond geographic locality to confirm the identification of others, as in the cases of *Zygaena romeo* – *Z*. *osterodensis*, *Melitaea athalia - M*. *nevadensis* and *Aricia agestis – A*. *artaxerxes*. It is interesting to note, however, that in certain rare cases DNA barcoding may give misleading results, for example, in cases of introgression between closely related species that are difficult or impossible to differentiate using morphological methods.

The sequencing of morphologically ambiguous populations, as well as museum specimens representing those populations and previously identified only using morphological methods, will also allow for improved species distribution mapping, imperative to understanding how populations shift and change through time. The DNA barcode may also provide a useful tool for the identification of preimaginal stages, sometimes difficult to identify using morphological criteria alone. Finally, the barcode provides a means of understanding how genetic diversity is distributed in Switzerland, thereby contributing to the development of conservation strategies that target biodiversity at multiple levels – morphological, ecological and genetic.

## Supporting information

S1 Fig Neighbor joining (NJ) tree based on DNA barcodes for all specimens sequenced for this study. (PDF)

S2 Fig Para- and polyphyly within the *Erebia tyndarus* complex. NJ tree based on DNA barcodes for specimens of the *Erebia tyndarus* complex present on BOLD. Specimens sequenced for this study are shown in blue. All specimens are presented with the names they have been given on BOLD, i.e. no names have been updated or otherwise modified. The DNA barcode cannot distinguish the three members of the *E*. *tyndarus* complex in Switzerland or in Europe.
(PDF)

S3 Fig Para- and polyphyly within the *Pyrgus alveus* complex. NJ tree based on DNA barcodes for specimens of the *Pyrgus alveus* complex present on BOLD. Specimens sequenced for this study are shown in blue. All specimens are presented with the names they have been given on BOLD, i.e. no names have been updated or otherwise modified. The DNA barcode cannot distinguish the three members of the *P*. *alveus* complex in Switzerland or in Europe.
(PDF)

S4 Fig *Melitaea athalia* and *M*. *nevadensis* form reciprocally monophyletic barcode clusters. NJ tree based on DNA barcodes for specimens of *Melitaea athalia* and *M*. *nevadensis* present on BOLD. Specimens sequenced for this study are shown in blue. All specimens are presented with the names they have been given on BOLD, i.e. no names have been updated or otherwise modified. Although *M*. *nevadensis* is more closely related to *M*. *deione* than *M*. *athalia* in NJ analyses, for the sake of brevity only specimens representing *M*. *athalia* and *M*. *nevadensis* are shown here. Numbers above certain nodes represent NJ bootstrap values above 50% based on 100 bootstrap replicates performed in PAUP*. There is no evidence of mitochondrial introgression from either taxon into the other.
(PDF)

S5 Fig Deep mitochondrial divergences in *Thymelicus lineola*. NJ tree based on DNA barcodes for specimens of *Thymelicus lineola* present on BOLD. Specimens sequenced for this study are shown in blue. All specimens are presented with the names they have been given on BOLD, i.e. no names have been updated or otherwise modified. Numbers above certain nodes represent NJ bootstrap values above 50% based on 100 bootstrap replicates performed in PAUP*. Barcoded specimens form five monophyletic clusters, one corresponding to a single specimen form Turkey; a second corresponding to two individuals, one from Spain and one from Germany; the third and fourth corresponding exclusively to haplotypes from European populations and a fifth to specimens sampled from both Europe and North America. North American individuals are most similar to certain populations from Switzerland, France, Germany, Austria and Spain, suggesting a possible source population for this species accidentally introduced into Canada in the early 20^th^ century.
(PDF)

S6 Fig Deep mitochondrial divergences in *Zygaena filipendulae*. NJ tree based on DNA barcodes for specimens of *Zygaena filipendulae* present on BOLD. Specimens sequenced for this study are shown in blue. All specimens are presented with the names they have been given on BOLD, i.e. no names have been updated or otherwise modified. Numbers above certain nodes represent NJ bootstrap values above 50% based on 100 bootstrap replicates performed in PAUP*. Barcoded specimens form two reciprocally monophyletic clusters.
(PDF)

S7 Fig Analysis of European DNA barcodes for *Erebia pronoe vergy and E*. *p*. *psathura*. NJ tree based on DNA barcodes for specimens of *Erebia pronoe* present on BOLD. Specimens sequenced for this study are shown in blue. All specimens are presented with the names they have been given on BOLD, i.e. no names have been updated or otherwise modified. Numbers above certain nodes represent NJ bootstrap values above 50% based on 100 bootstrap replicates performed in PAUP*. Elevating *E*. *p*. *vergy and E*. *p*. *psathura* to species level based on the results of our analyses of Swiss specimens would render *E*. *pronoe* polyphyletic and would have taxonomic implications for other European populations.
(PDF)

S8 Fig *Lycaena tityrus tityrus* and *L*. *t*. *subalpina* form reciprocally monophyletic barcode clusters. NJ tree based on DNA barcodes for specimens of *Lycaena tityrus* present on BOLD. Specimens sequenced for this study are shown in blue. All specimens are presented with the names they have been given on BOLD, i.e. no names have been updated or otherwise modified. Numbers above certain nodes represent NJ bootstrap values above 50% based on 100 bootstrap replicates performed in PAUP*. In cases where individuals on BOLD have only been identified to species, subspecies have been inferred based on locality. This inference suggests that *Lycaena tityrus tityrus* and *L*. *t*. *subalpina* form reciprocally monophyletic barcode clusters.
(PDF)

S9 Fig Swiss populations of *Melitaea deione a*re unique in the western Palaearctic. NJ tree based on DNA barcodes for specimens of *Melitaea deione* present on BOLD. Specimens sequenced for this study, representing the Swiss subspecies *M*. *d*. *berisalii*, are shown in blue. Numbers above certain nodes represent NJ bootstrap values above 50% based on 100 bootstrap replicates performed in PAUP*. Individuals of *M*. *d*. *berisalii* represent a unique, independently evolving mitochondrial lineage, providing support for the endemic status of Swiss populations of *Melitaea deione berisalii*.
(PDF)

S10 Fig Swiss populations of *Kretania trappi* are unique compared to Italian populations from the Alto Adige–Sudtirol. NJ tree based on DNA barcodes for specimens of *Kretania trappi* present on BOLD. Specimens sequenced for this study are shown in blue. All specimens are presented with the names they have been given on BOLD, i.e. no names have been updated or otherwise modified. Numbers above certain nodes represent NJ bootstrap values above 50% based on 100 bootstrap replicates performed in PAUP*. A Swiss specimen of *K*. *trappi* represents a unique mitochondrial haplotype compared to Italian populations from the Alto Adige – Sudtirol, providing preliminary support for the endemic status of Swiss populations of *K*. *trappi*.
(PDF)

S1 Table Specimen list. Complete list of 868 specimens sequenced for this study, including collection information, unique specimen identifiers, associated CO1 sequences with BOLD process ID numbers and specimen deposition information.
(XLSX)

S2 Table Reference species list. Two-hundred twenty-four species were considered as Swiss residents for the purposes of this study. All changes from the Swiss Red List (Wermeille et al. 2014) have been noted. Species for which no sequences were obtained are marked in red.
(XLSX)
