# Supplementary figures and images for: A DNA barcode reference library for Swiss butterflies and forester moths as a tool for species identification, systematics and conservation
Source: PLoS One. 2018 Dec 21;13(12):e0208639. doi: 10.1371/journal.pone.0208639 (PMC6303096; doi:10.1371/journal.pone.0208639)

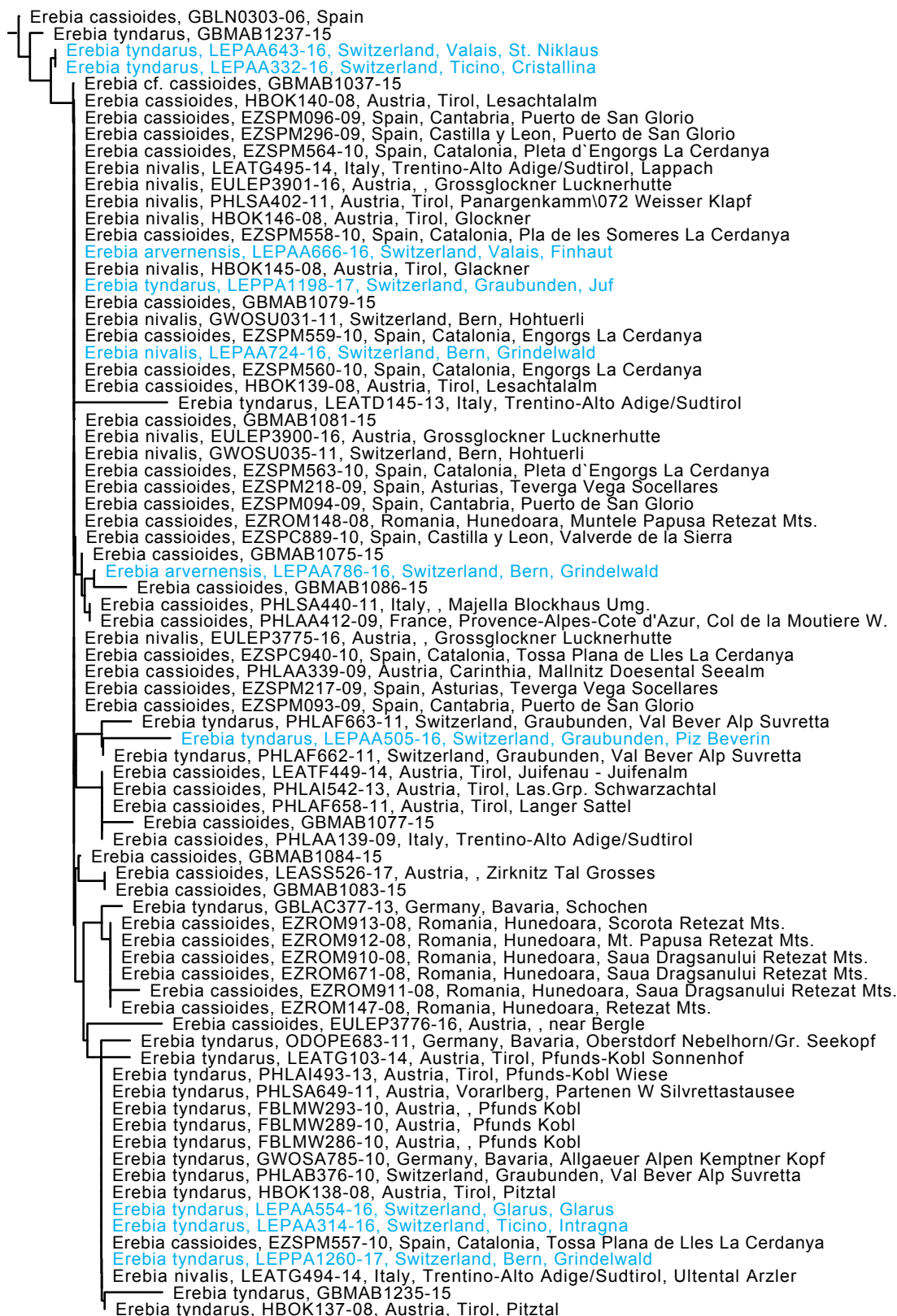

Supplement: S2 Fig — NJ tree based on DNA barcodes for specimens of the Erebia tyndarus complex present on BOLD. Specimens sequenced for this study are shown in blue. All specimens are presented with the names they have been given on BOLD, i.e. no names have been updated or otherwise modified. The DNA barcode cannot distinguish the three members of the E. tyndarus complex in Switzerland or in Europe. (PDF) [file pone.0208639.s002.pdf]

Fig. S4

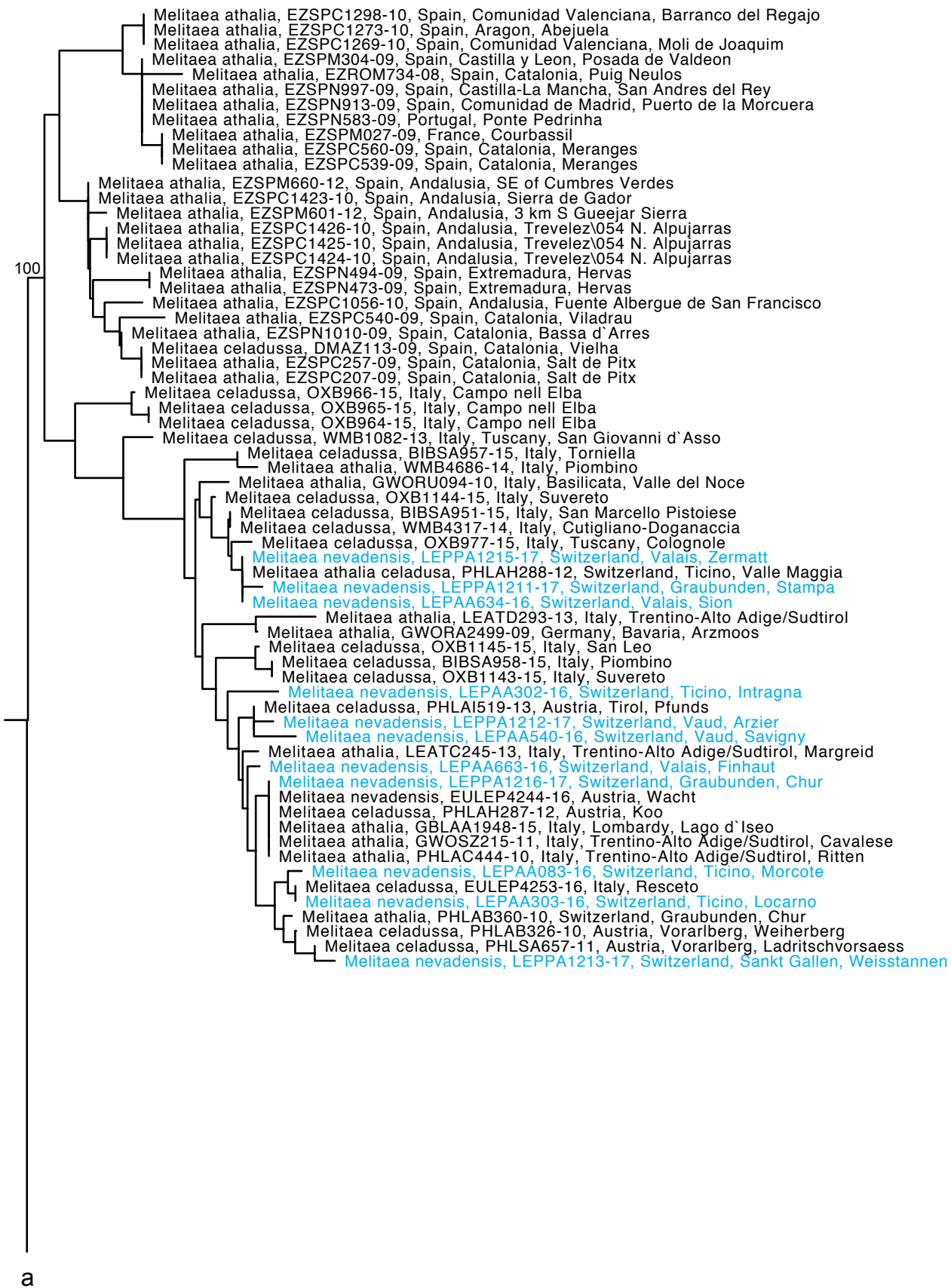

Fig. S4 (cont'd)

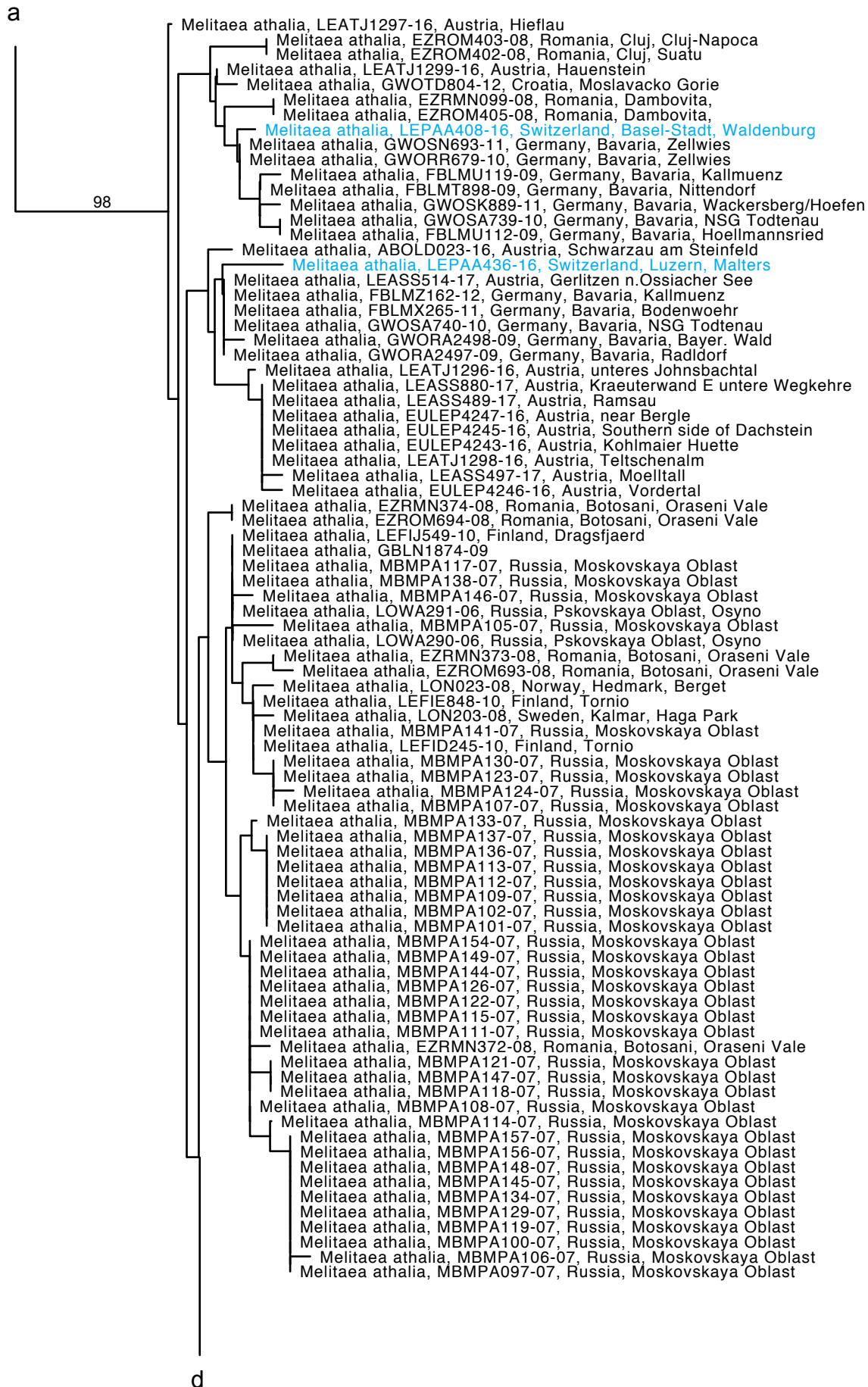

Fig. S4 (cont'd)

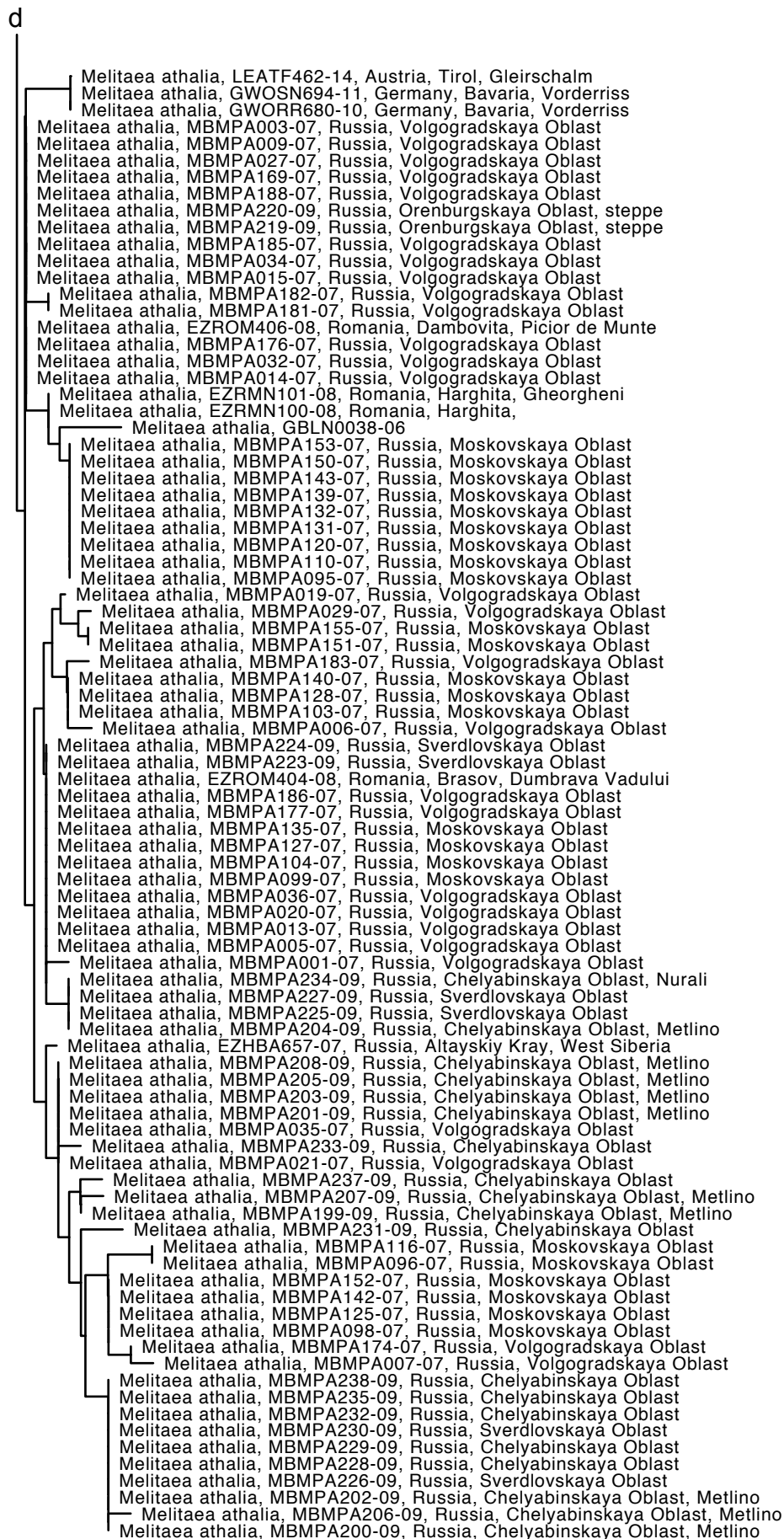

Supplement: S4 Fig — NJ tree based on DNA barcodes for specimens of Melitaea athalia and M. nevadensis present on BOLD. Specimens sequenced for this study are shown in blue. All specimens are presented with the names they have been given on BOLD, i.e. no names have been updated or otherwise modified. Although M. nevadensis is more closely related to M. deione than M. athalia in NJ analyses, for the sake of brevity only specimens representing M. athalia and M. nevadensis are shown here. Numbers above certain nodes represent NJ bootstrap values above 50% based on 100 bootstrap replicates performed in PAUP*. There is no evidence of mitochondrial introgression from either taxon into the other. (PDF) [file pone.0208639.s004.pdf]

Fig. S6

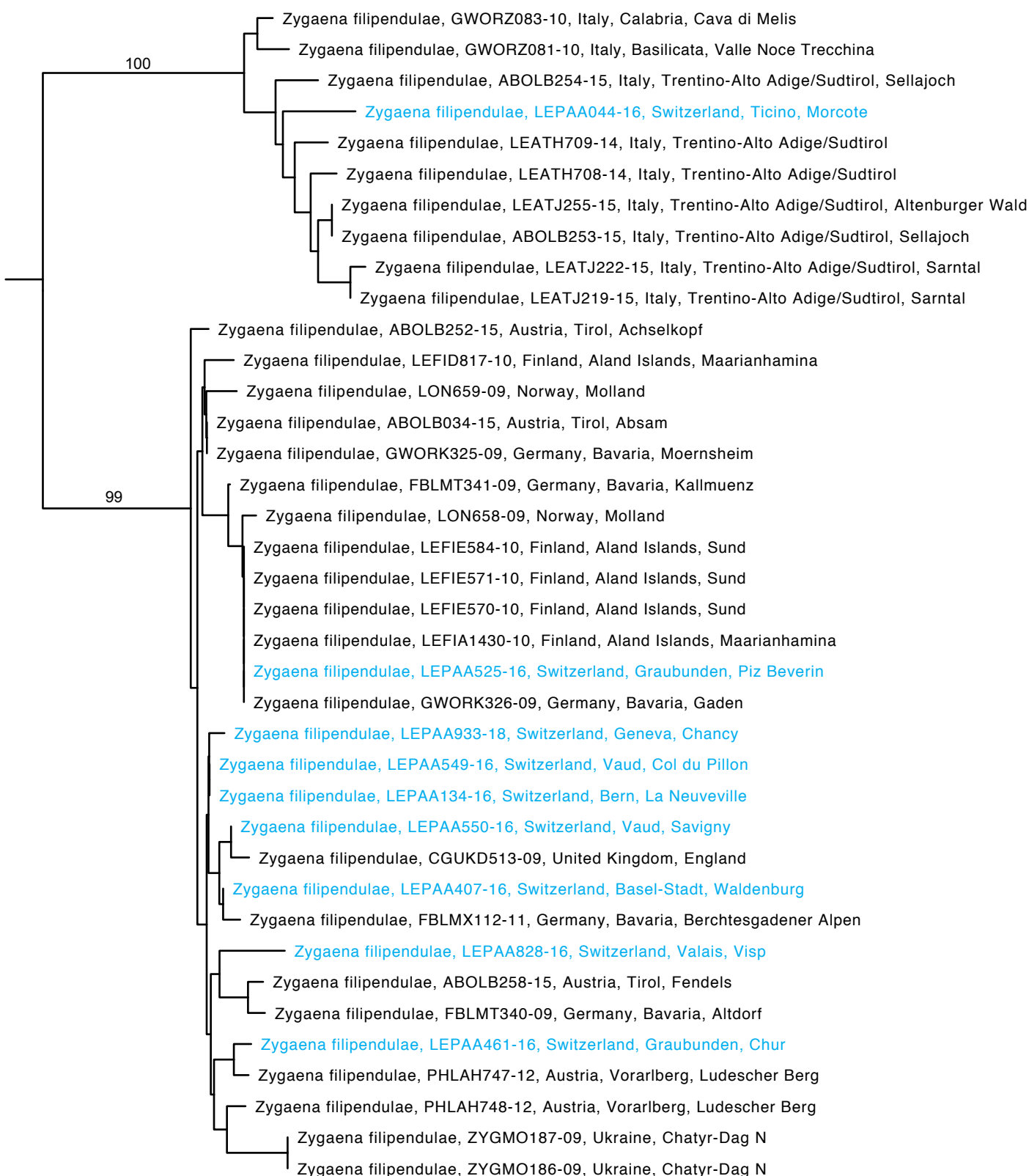

0.020

Supplement: S6 Fig — NJ tree based on DNA barcodes for specimens of Zygaena filipendulae present on BOLD. Specimens sequenced for this study are shown in blue. All specimens are presented with the names they have been given on BOLD, i.e. no names have been updated or otherwise modified. Numbers above certain nodes represent NJ bootstrap values above 50% based on 100 bootstrap replicates performed in PAUP*. Barcoded specimens form two reciprocally monophyletic clusters. (PDF) [file pone.0208639.s006.pdf]

Fig S7

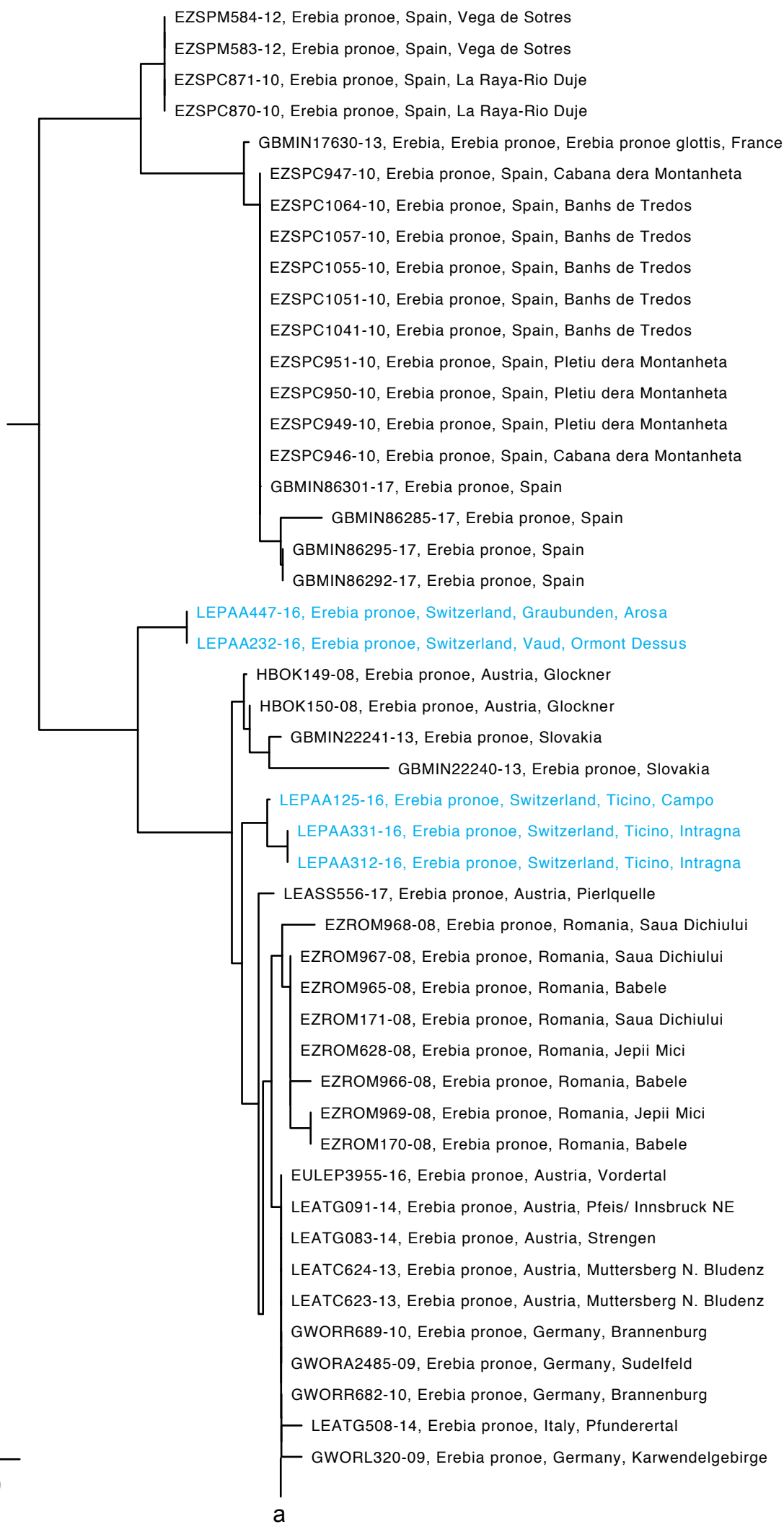

Fig S7 (cont'd)

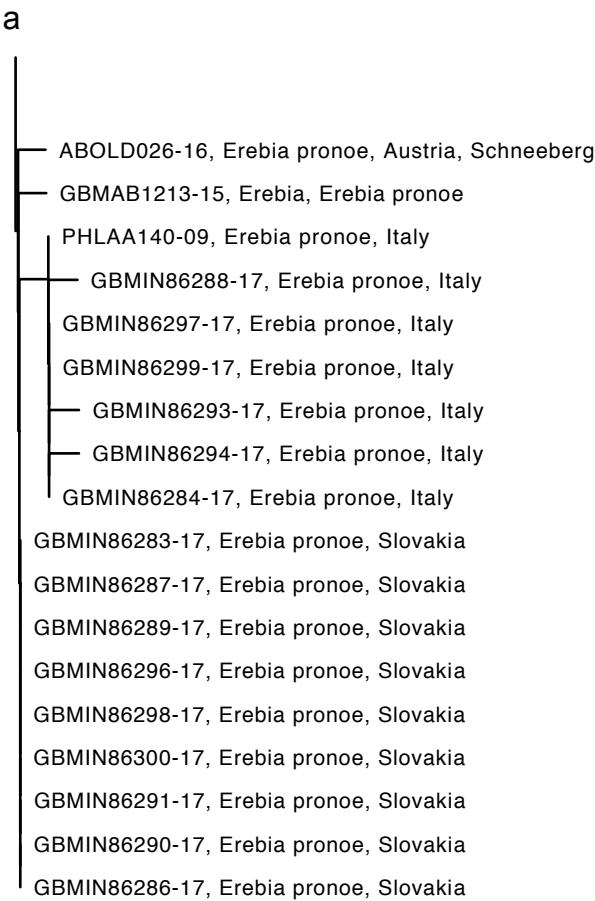

Supplement: S7 Fig — NJ tree based on DNA barcodes for specimens of Erebia pronoe present on BOLD. Specimens sequenced for this study are shown in blue. All specimens are presented with the names they have been given on BOLD, i.e. no names have been updated or otherwise modified. Numbers above certain nodes represent NJ bootstrap values above 50% based on 100 bootstrap replicates performed in PAUP*. Elevating E. p. vergy and E. p. psathura to species level based on the results of our analyses of Swiss specimens would render E. pronoe polyphyletic and would have taxonomic implications for other European populations. (PDF) [file pone.0208639.s007.pdf]

Fig. S8

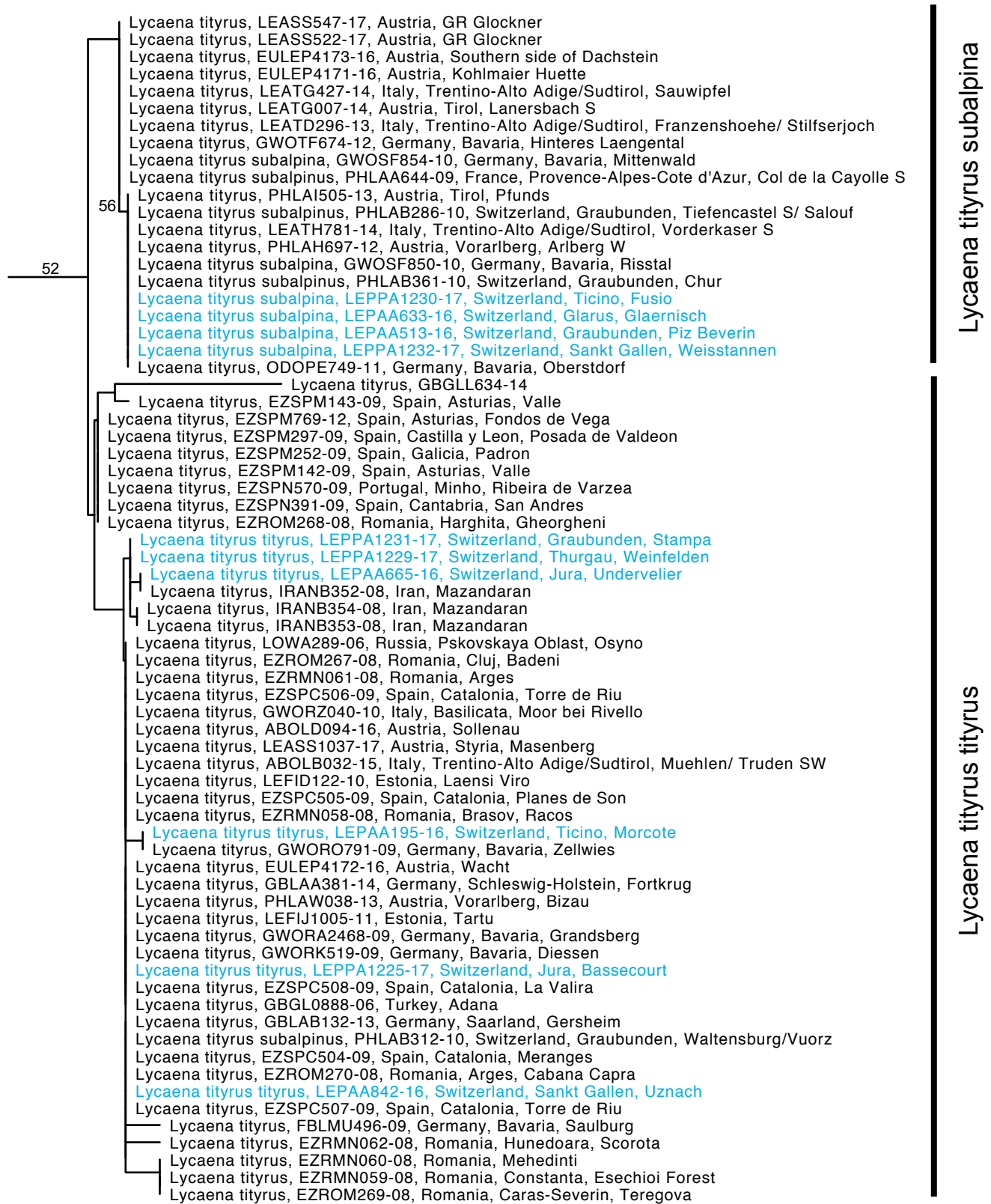

Supplement: S8 Fig — NJ tree based on DNA barcodes for specimens of Lycaena tityrus present on BOLD. Specimens sequenced for this study are shown in blue. All specimens are presented with the names they have been given on BOLD, i.e. no names have been updated or otherwise modified. Numbers above certain nodes represent NJ bootstrap values above 50% based on 100 bootstrap replicates performed in PAUP*. In cases where individuals on BOLD have only been identified to species, subspecies have been inferred based on locality. This inference suggests that Lycaena tityrus tityrus and L. t. subalpina form reciprocally monophyletic barcode clusters. (PDF) [file pone.0208639.s008.pdf]

Fig. S9

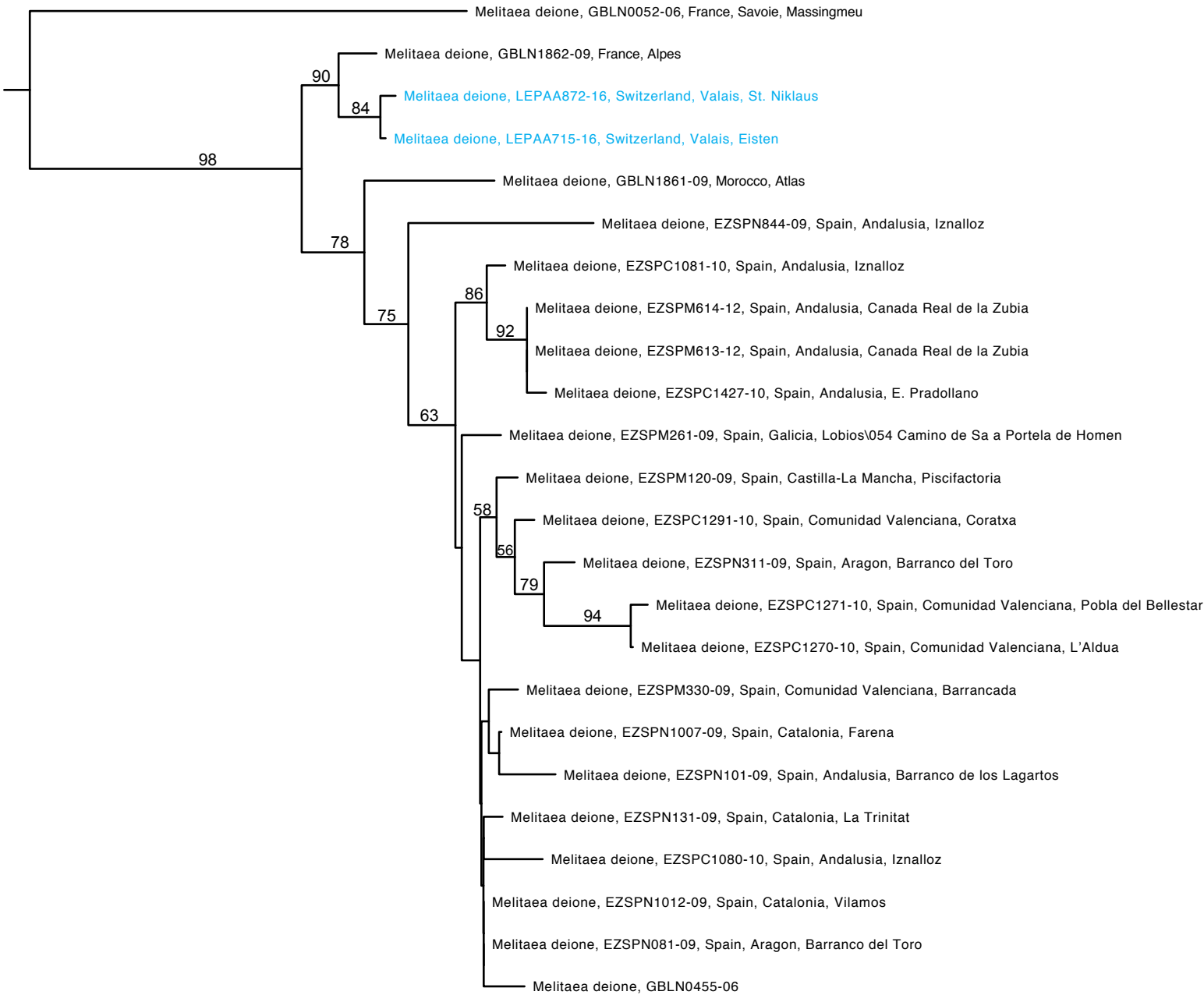

0.0080

Supplement: S9 Fig — NJ tree based on DNA barcodes for specimens of Melitaea deione present on BOLD. Specimens sequenced for this study, representing the Swiss subspecies M. d. berisalii, are shown in blue. Numbers above certain nodes represent NJ bootstrap values above 50% based on 100 bootstrap replicates performed in PAUP*. Individuals of M. d. berisalii represent a unique, independently evolving mitochondrial lineage, providing support for the endemic status of Swiss populations of Melitaea deione berisalii. (PDF) [file pone.0208639.s009.pdf]

Fig. S10

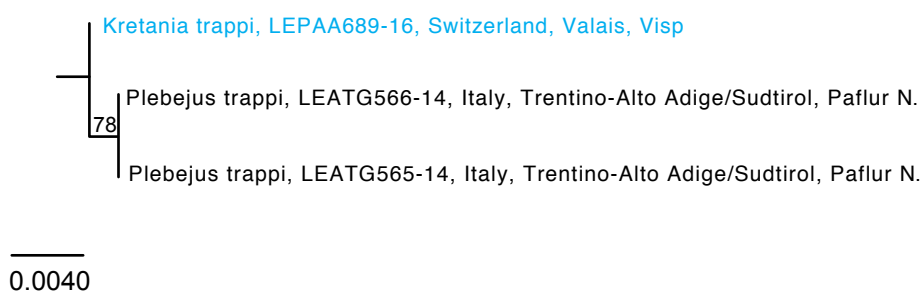

Supplement: S10 Fig — NJ tree based on DNA barcodes for specimens of Kretania trappi present on BOLD. Specimens sequenced for this study are shown in blue. All specimens are presented with the names they have been given on BOLD, i.e. no names have been updated or otherwise modified. Numbers above certain nodes represent NJ bootstrap values above 50% based on 100 bootstrap replicates performed in PAUP*. A Swiss specimen of K. trappi represents a unique mitochondrial haplotype compared to Italian populations from the Alto Adige – Sudtirol, providing preliminary support for the endemic status of Swiss populations of K. trappi. (PDF) [file pone.0208639.s010.pdf]
