# Supplementary material for: A DNA barcode reference library for Swiss butterflies and forester moths as a tool for species identification, systematics and conservation
Source: PLoS One. 2018 Dec 21;13(12):e0208639. doi: 10.1371/journal.pone.0208639 (PMC6303096; doi:10.1371/journal.pone.0208639)
Supplement: S3 Fig — NJ tree based on DNA barcodes for specimens of the Pyrgus alveus complex present on BOLD. Specimens sequenced for this study are shown in blue. All specimens are presented with the names they have been given on BOLD, i.e. no names have been updated or otherwise modified. The DNA barcode cannot distinguish the three members of the P. alveus complex in Switzerland or in Europe. (PDF) [file pone.0208639.s003.pdf]

Fig. S3

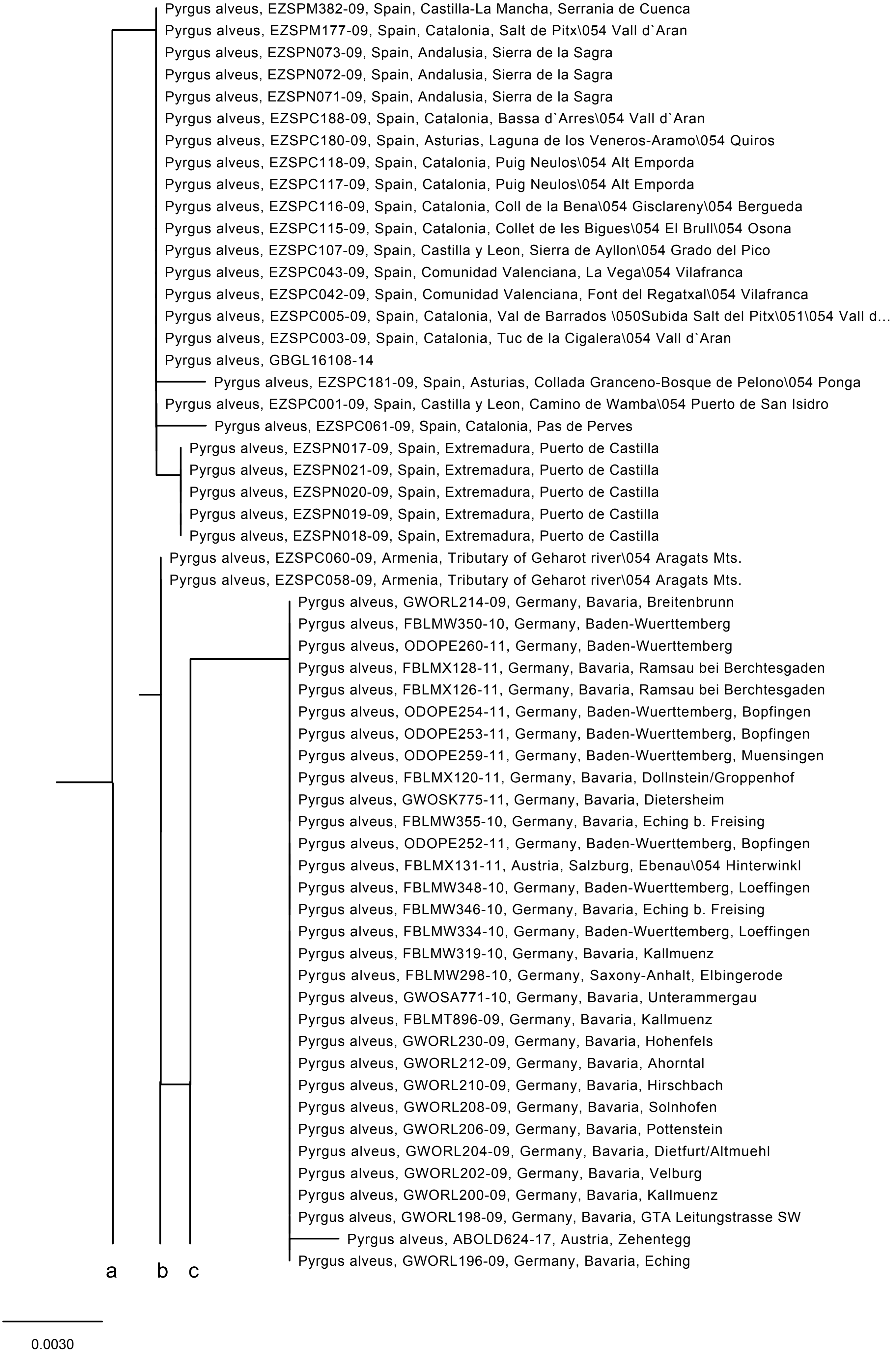

Pyrgus alveus, EZSPM382-09, Spain, Castilla-La Mancha, Serrania de Cuenca
Pyrgus alveus, EZSPM177-09, Spain, Catalonia, Salt de Pitx\054 Vall d`Aran
Pyrgus alveus, EZSPN073-09, Spain, Andalusia, Sierra de la Sagra
Pyrgus alveus, EZSPN072-09, Spain, Andalusia, Sierra de la Sagra
Pyrgus alveus, EZSPN071-09, Spain, Andalusia, Sierra de la Sagra
Pyrgus alveus, EZSPC188-09, Spain, Catalonia, Bassa d`Arres\054 Vall d`Aran
Pyrgus alveus, EZSPC180-09, Spain, Asturias, Laguna de los Veneros-Aramo\054 Quiros
Pyrgus alveus, EZSPC118-09, Spain, Catalonia, Puig Neulos\054 Alt Emporda
Pyrgus alveus, EZSPC117-09, Spain, Catalonia, Puig Neulos\054 Alt Emporda
Pyrgus alveus, EZSPC116-09, Spain, Catalonia, Coll de la Bena\054 Gisclareny\054 Bergueda
Pyrgus alveus, EZSPC115-09, Spain, Catalonia, Collet de les Bigues\054 El Brull\054 Osona
Pyrgus alveus, EZSPC107-09, Spain, Castilla y Leon, Sierra de Ayllon\054 Grado del Pico
Pyrgus alveus, EZSPC043-09, Spain, Comunidad Valenciana, La Vega\054 Vilafranca
Pyrgus alveus, EZSPC042-09, Spain, Comunidad Valenciana, Font del Regatxal\054 Vilafranca
Pyrgus alveus, EZSPC005-09, Spain, Catalonia, Val de Barrados \050Subida Salt del Pitx\051\054 Vall d...
Pyrgus alveus, EZSPC003-09, Spain, Catalonia, Tuc de la Cigalera\054 Vall d`Aran
Pyrgus alveus, GBGL16108-14
Pyrgus alveus, EZSPC181-09, Spain, Asturias, Collada Granceno-Bosque de Pelono\054 Ponga
Pyrgus alveus, EZSPC001-09, Spain, Castilla y Leon, Camino de Wamba\054 Puerto de San Isidro
Pyrgus alveus, EZSPC061-09, Spain, Catalonia, Pas de Perves
Pyrgus alveus, EZSPN017-09, Spain, Extremadura, Puerto de Castilla
Pyrgus alveus, EZSPN021-09, Spain, Extremadura, Puerto de Castilla
Pyrgus alveus, EZSPN020-09, Spain, Extremadura, Puerto de Castilla
Pyrgus alveus, EZSPN019-09, Spain, Extremadura, Puerto de Castilla
Pyrgus alveus, EZSPN018-09, Spain, Extremadura, Puerto de Castilla
Pyrgus alveus, EZSPC060-09, Armenia, Tributary of Geharot river\054 Aragats Mts.
Pyrgus alveus, EZSPC058-09, Armenia, Tributary of Geharot river\054 Aragats Mts.
Pyrgus alveus, GWORL214-09, Germany, Bavaria, Breitenbrunn
Pyrgus alveus, FBLMW350-10, Germany, Baden-Wuerttemberg
Pyrgus alveus, ODOPE260-11, Germany, Baden-Wuerttemberg
Pyrgus alveus, FBLMX128-11, Germany, Bavaria, Ramsau bei Berchtesgaden
Pyrgus alveus, FBLMX126-11, Germany, Bavaria, Ramsau bei Berchtesgaden
Pyrgus alveus, ODOPE254-11, Germany, Baden-Wuerttemberg, Bopfingen
Pyrgus alveus, ODOPE253-11, Germany, Baden-Wuerttemberg, Bopfingen
Pyrgus alveus, ODOPE259-11, Germany, Baden-Wuerttemberg, Muensingen
Pyrgus alveus, FBLMX120-11, Germany, Bavaria, Dollnstein/Groppenhof
Pyrgus alveus, GWOSK775-11, Germany, Bavaria, Dietersheim
Pyrgus alveus, FBLMW355-10, Germany, Bavaria, Eching b. Freising
Pyrgus alveus, ODOPE252-11, Germany, Baden-Wuerttemberg, Bopfingen
Pyrgus alveus, FBLMX131-11, Austria, Salzburg, Ebenau\054 Hinterwinkl
Pyrgus alveus, FBLMW348-10, Germany, Baden-Wuerttemberg, Loeffingen
Pyrgus alveus, FBLMW346-10, Germany, Bavaria, Eching b. Freising
Pyrgus alveus, FBLMW334-10, Germany, Baden-Wuerttemberg, Loeffingen
Pyrgus alveus, FBLMW319-10, Germany, Bavaria, Kallmuenz
Pyrgus alveus, FBLMW298-10, Germany, Saxony-Anhalt, Elbingerode
Pyrgus alveus, GWOSA771-10, Germany, Bavaria, Unterammergau
Pyrgus alveus, FBLMT896-09, Germany, Bavaria, Kallmuenz
Pyrgus alveus, GWORL230-09, Germany, Bavaria, Hohenfels
Pyrgus alveus, GWORL212-09, Germany, Bavaria, Ahorntal
Pyrgus alveus, GWORL210-09, Germany, Bavaria, Hirschbach
Pyrgus alveus, GWORL208-09, Germany, Bavaria, Solnhofen
Pyrgus alveus, GWORL206-09, Germany, Bavaria, Pottenstein
Pyrgus alveus, GWORL204-09, Germany, Bavaria, Dietfurt/Altmuehl
Pyrgus alveus, GWORL202-09, Germany, Bavaria, Velburg
Pyrgus alveus, GWORL200-09, Germany, Bavaria, Kallmuenz
Pyrgus alveus, GWORL198-09, Germany, Bavaria, GTA Leitungstrasse SW
Pyrgus alveus, ABOLD624-17, Austria, Zehentegg
Pyrgus alveus, GWORL196-09, Germany, Bavaria, Eching
a
b
c
0.0030

Fig. S3 (cont'd)

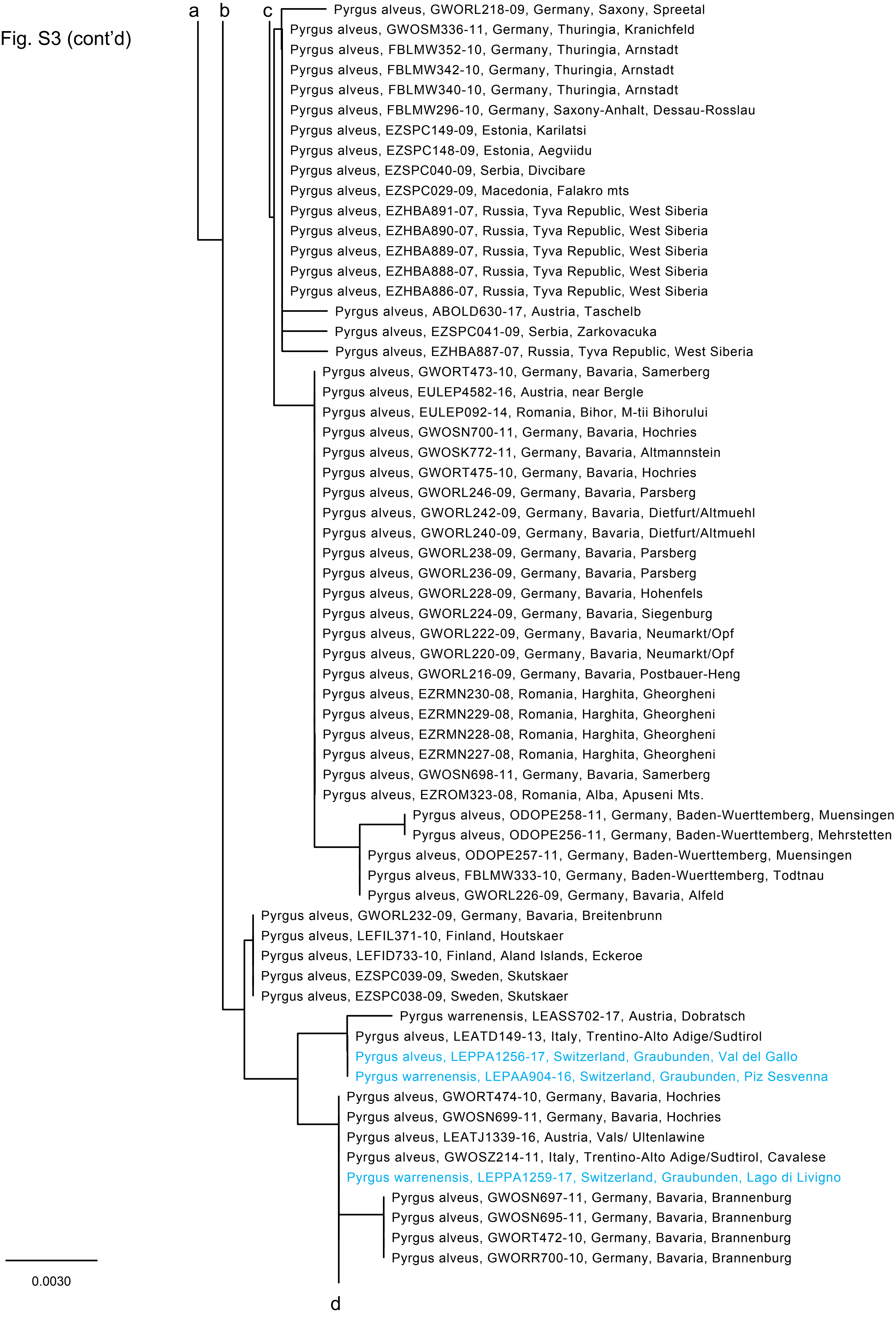
a
b
c
Pyrgus alveus, GWORL218-09, Germany, Saxony, Spreetal
Pyrgus alveus, GWOSM336-11, Germany, Thuringia, Kranichfeld
Pyrgus alveus, FBLMW352-10, Germany, Thuringia, Arnstadt
Pyrgus alveus, FBLMW342-10, Germany, Thuringia, Arnstadt
Pyrgus alveus, FBLMW340-10, Germany, Thuringia, Arnstadt
Pyrgus alveus, FBLMW296-10, Germany, Saxony-Anhalt, Dessau-Rosslau
Pyrgus alveus, EZSPC149-09, Estonia, Karilatsi
Pyrgus alveus, EZSPC148-09, Estonia, Aegviidu
Pyrgus alveus, EZSPC040-09, Serbia, Divcibare
Pyrgus alveus, EZSPC029-09, Macedonia, Falakro mts
Pyrgus alveus, EZHBA891-07, Russia, Tyva Republic, West Siberia
Pyrgus alveus, EZHBA890-07, Russia, Tyva Republic, West Siberia
Pyrgus alveus, EZHBA889-07, Russia, Tyva Republic, West Siberia
Pyrgus alveus, EZHBA888-07, Russia, Tyva Republic, West Siberia
Pyrgus alveus, EZHBA886-07, Russia, Tyva Republic, West Siberia
Pyrgus alveus, ABOLD630-17, Austria, Taschelb
Pyrgus alveus, EZSPC041-09, Serbia, Zarkovacuka
Pyrgus alveus, EZHBA887-07, Russia, Tyva Republic, West Siberia
Pyrgus alveus, GWORT473-10, Germany, Bavaria, Samerberg
Pyrgus alveus, EULEP4582-16, Austria, near Bergle
Pyrgus alveus, EULEP092-14, Romania, Bihor, M-tii Bihorului
Pyrgus alveus, GWOSN700-11, Germany, Bavaria, Hochries
Pyrgus alveus, GWOSK772-11, Germany, Bavaria, Altmannstein
Pyrgus alveus, GWORT475-10, Germany, Bavaria, Hochries
Pyrgus alveus, GWORL246-09, Germany, Bavaria, Parsberg
Pyrgus alveus, GWORL242-09, Germany, Bavaria, Dietfurt/Altmuehl
Pyrgus alveus, GWORL240-09, Germany, Bavaria, Dietfurt/Altmuehl
Pyrgus alveus, GWORL238-09, Germany, Bavaria, Parsberg
Pyrgus alveus, GWORL236-09, Germany, Bavaria, Parsberg
Pyrgus alveus, GWORL228-09, Germany, Bavaria, Hohenfels
Pyrgus alveus, GWORL224-09, Germany, Bavaria, Siegenburg
Pyrgus alveus, GWORL222-09, Germany, Bavaria, Neumarkt/Opf
Pyrgus alveus, GWORL220-09, Germany, Bavaria, Neumarkt/Opf
Pyrgus alveus, GWORL216-09, Germany, Bavaria, Postbauer-Heng
Pyrgus alveus, EZRMN230-08, Romania, Harghita, Gheorgheni
Pyrgus alveus, EZRMN229-08, Romania, Harghita, Gheorgheni
Pyrgus alveus, EZRMN228-08, Romania, Harghita, Gheorgheni
Pyrgus alveus, EZRMN227-08, Romania, Harghita, Gheorgheni
Pyrgus alveus, GWOSN698-11, Germany, Bavaria, Samerberg
Pyrgus alveus, EZROM323-08, Romania, Alba, Apuseni Mts.
Pyrgus alveus, ODOPE258-11, Germany, Baden-Wuerttemberg, Muensingen
Pyrgus alveus, ODOPE256-11, Germany, Baden-Wuerttemberg, Mehrstetten
Pyrgus alveus, ODOPE257-11, Germany, Baden-Wuerttemberg, Muensingen
Pyrgus alveus, FBLMW333-10, Germany, Baden-Wuerttemberg, Todtnau
Pyrgus alveus, GWORL226-09, Germany, Bavaria, Alfeld
Pyrgus alveus, GWORL232-09, Germany, Bavaria, Breitenbrunn
Pyrgus alveus, LEFIL371-10, Finland, Houtskaer
Pyrgus alveus, LEFID733-10, Finland, Aland Islands, Eckeroe
Pyrgus alveus, EZSPC039-09, Sweden, Skutskaer
Pyrgus alveus, EZSPC038-09, Sweden, Skutskaer
Pyrgus warrenensis, LEASS702-17, Austria, Dobratsch
Pyrgus alveus, LEATD149-13, Italy, Trentino-Alto Adige/Sudtirol
Pyrgus alveus, LEPPA1256-17, Switzerland, Graubunden, Val del Gallo
Pyrgus warrenensis, LEPAA904-16, Switzerland, Graubunden, Piz Sesvenna
Pyrgus alveus, GWORT474-10, Germany, Bavaria, Hochries
Pyrgus alveus, GWOSN699-11, Germany, Bavaria, Hochries
Pyrgus alveus, LEATJ1339-16, Austria, Vals/ Ultenlawine
Pyrgus alveus, GWOSZ214-11, Italy, Trentino-Alto Adige/Sudtirol, Cavalese
Pyrgus warrenensis, LEPPA1259-17, Switzerland, Graubunden, Lago di Livigno
Pyrgus alveus, GWOSN697-11, Germany, Bavaria, Brannenburg
Pyrgus alveus, GWOSN695-11, Germany, Bavaria, Brannenburg
Pyrgus alveus, GWORT472-10, Germany, Bavaria, Brannenburg
Pyrgus alveus, GWORR700-10, Germany, Bavaria, Brannenburg
0.0030
d

Fig. S3 (cont'd)

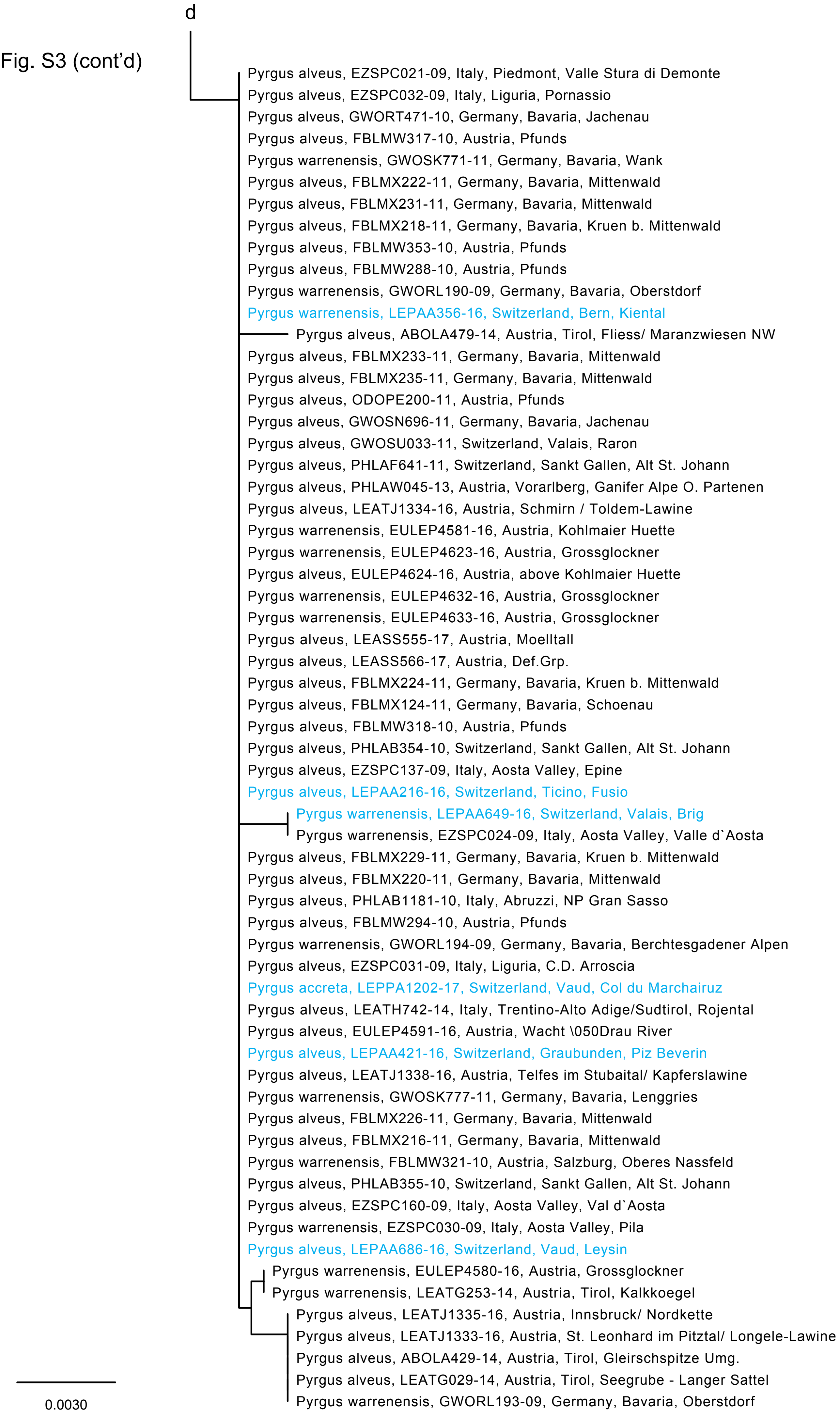
d
Pyrgus alveus, EZSPC021-09, Italy, Piedmont, Valle Stura di Demonte
Pyrgus alveus, EZSPC032-09, Italy, Liguria, Pornassio
Pyrgus alveus, GWORT471-10, Germany, Bavaria, Jachenau
Pyrgus alveus, FBLMW317-10, Austria, Pfunds
Pyrgus warrenensis, GWOSK771-11, Germany, Bavaria, Wank
Pyrgus alveus, FBLMX222-11, Germany, Bavaria, Mittenwald
Pyrgus alveus, FBLMX231-11, Germany, Bavaria, Mittenwald
Pyrgus alveus, FBLMX218-11, Germany, Bavaria, Kruen b. Mittenwald
Pyrgus alveus, FBLMW353-10, Austria, Pfunds
Pyrgus alveus, FBLMW288-10, Austria, Pfunds
Pyrgus warrenensis, GWORL190-09, Germany, Bavaria, Oberstdorf
Pyrgus warrenensis, LEPAA356-16, Switzerland, Bern, Kiental
Pyrgus alveus, ABOLA479-14, Austria, Tirol, Fliess/ Maranzwiesen NW
Pyrgus alveus, FBLMX233-11, Germany, Bavaria, Mittenwald
Pyrgus alveus, FBLMX235-11, Germany, Bavaria, Mittenwald
Pyrgus alveus, ODOPE200-11, Austria, Pfunds
Pyrgus alveus, GWOSN696-11, Germany, Bavaria, Jachenau
Pyrgus alveus, GWOSU033-11, Switzerland, Valais, Raron
Pyrgus alveus, PHLAF641-11, Switzerland, Sankt Gallen, Alt St. Johann
Pyrgus alveus, PHLAW045-13, Austria, Vorarlberg, Ganifer Alpe O. Partenen
Pyrgus alveus, LEATJ1334-16, Austria, Schmirn / Toldem-Lawine
Pyrgus warrenensis, EULEP4581-16, Austria, Kohlmaier Huette
Pyrgus warrenensis, EULEP4623-16, Austria, Grossglockner
Pyrgus alveus, EULEP4624-16, Austria, above Kohlmaier Huette
Pyrgus warrenensis, EULEP4632-16, Austria, Grossglockner
Pyrgus warrenensis, EULEP4633-16, Austria, Grossglockner
Pyrgus alveus, LEASS555-17, Austria, Moelltall
Pyrgus alveus, LEASS566-17, Austria, Def.Grp.
Pyrgus alveus, FBLMX224-11, Germany, Bavaria, Kruen b. Mittenwald
Pyrgus alveus, FBLMX124-11, Germany, Bavaria, Schoenau
Pyrgus alveus, FBLMW318-10, Austria, Pfunds
Pyrgus alveus, PHLAB354-10, Switzerland, Sankt Gallen, Alt St. Johann
Pyrgus alveus, EZSPC137-09, Italy, Aosta Valley, Epine
Pyrgus alveus, LEPAA216-16, Switzerland, Ticino, Fusio
Pyrgus warrenensis, LEPAA649-16, Switzerland, Valais, Brig
Pyrgus warrenensis, EZSPC024-09, Italy, Aosta Valley, Valle d`Aosta
Pyrgus alveus, FBLMX229-11, Germany, Bavaria, Kruen b. Mittenwald
Pyrgus alveus, FBLMX220-11, Germany, Bavaria, Mittenwald
Pyrgus alveus, PHLAB1181-10, Italy, Abruzzi, NP Gran Sasso
Pyrgus alveus, FBLMW294-10, Austria, Pfunds
Pyrgus warrenensis, GWORL194-09, Germany, Bavaria, Berchtesgadener Alpen
Pyrgus alveus, EZSPC031-09, Italy, Liguria, C.D. Arroscia
Pyrgus accreta, LEPPA1202-17, Switzerland, Vaud, Col du Marchairuz
Pyrgus alveus, LEATH742-14, Italy, Trentino-Alto Adige/Sudtirol, Rojental
Pyrgus alveus, EULEP4591-16, Austria, Wacht \050Drau River
Pyrgus alveus, LEPAA421-16, Switzerland, Graubunden, Piz Beverin
Pyrgus alveus, LEATJ1338-16, Austria, Telfes im Stubaital/ Kapferslawine
Pyrgus warrenensis, GWOSK777-11, Germany, Bavaria, Lenggries
Pyrgus alveus, FBLMX226-11, Germany, Bavaria, Mittenwald
Pyrgus alveus, FBLMX216-11, Germany, Bavaria, Mittenwald
Pyrgus warrenensis, FBLMW321-10, Austria, Salzburg, Oberes Nassfeld
Pyrgus alveus, PHLAB355-10, Switzerland, Sankt Gallen, Alt St. Johann
Pyrgus alveus, EZSPC160-09, Italy, Aosta Valley, Val d`Aosta
Pyrgus warrenensis, EZSPC030-09, Italy, Aosta Valley, Pila
Pyrgus alveus, LEPAA686-16, Switzerland, Vaud, Leysin
Pyrgus warrenensis, EULEP4580-16, Austria, Grossglockner
Pyrgus warrenensis, LEATG253-14, Austria, Tirol, Kalkkoegel
Pyrgus alveus, LEATJ1335-16, Austria, Innsbruck/ Nordkette
Pyrgus alveus, LEATJ1333-16, Austria, St. Leonhard im Pitztal/ Longele-Lawine
Pyrgus alveus, ABOLA429-14, Austria, Tirol, Gleirschspitze Umg.
Pyrgus alveus, LEATG029-14, Austria, Tirol, Seegrube - Langer Sattel
Pyrgus warrenensis, GWORL193-09, Germany, Bavaria, Oberstdorf
0.0030
