# Supplementary material for: A DNA barcode reference library for Swiss butterflies and forester moths as a tool for species identification, systematics and conservation
Source: PLoS One. 2018 Dec 21;13(12):e0208639. doi: 10.1371/journal.pone.0208639 (PMC6303096; doi:10.1371/journal.pone.0208639)

Fig S1

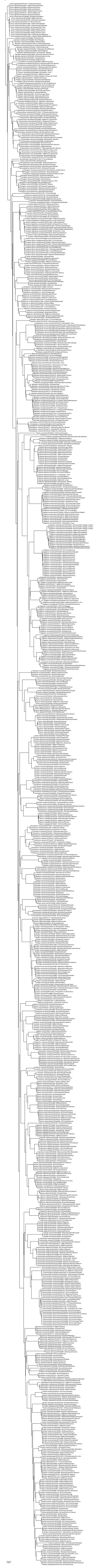

Supplement: S1 Fig — (PDF) [file pone.0208639.s001.pdf]
