# Supplementary material for: A DNA barcode reference library for Swiss butterflies and forester moths as a tool for species identification, systematics and conservation
Source: PLoS One. 2018 Dec 21;13(12):e0208639. doi: 10.1371/journal.pone.0208639 (PMC6303096; doi:10.1371/journal.pone.0208639)

Fig S5

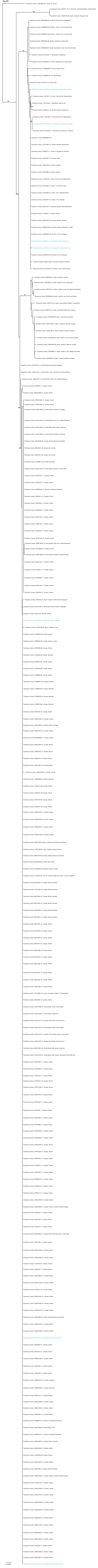

Supplement: S5 Fig — NJ tree based on DNA barcodes for specimens of Thymelicus lineola present on BOLD. Specimens sequenced for this study are shown in blue. All specimens are presented with the names they have been given on BOLD, i.e. no names have been updated or otherwise modified. Numbers above certain nodes represent NJ bootstrap values above 50% based on 100 bootstrap replicates performed in PAUP*. Barcoded specimens form five monophyletic clusters, one corresponding to a single specimen form Turkey; a second corresponding to two individuals, one from Spain and one from Germany; the third and fourth corresponding exclusively to haplotypes from European populations and a fifth to specimens sampled from both Europe and North America. North American individuals are most similar to certain populations from Switzerland, France, Germany, Austria and Spain, suggesting a possible source population for this species accidentally introduced into Canada in the early 20th century. (PDF) [file pone.0208639.s005.pdf]
